# Modified recombinant human IgG1‐Fc is superior to natural intravenous immunoglobulin at inhibiting immune‐mediated demyelination

**DOI:** 10.1111/imm.13341

**Published:** 2021-05-09

**Authors:** Christine Baksmeier, Pat Blundell, Julia Steckel, Verena Schultz, Quan Gu, Ana Da Silva Filipe, Alain Kohl, Chris Linnington, Dongli Lu, Anne Dell, Stuart Haslam, Jiabin Wang, Dan Czajkowsky, Norbert Goebels, Richard J. Pleass

**Affiliations:** ^1^ Department of Neurology Medical Faculty Heinrich‐Heine‐University Duesseldorf Duesseldorf Germany; ^2^ Department of Tropical Disease Biology Liverpool School of Tropical Medicine Liverpool UK; ^3^ Institute of Infection, Immunity and Inflammation College of Medical Veterinary and Life Sciences University of Glasgow Glasgow UK; ^4^ Department of Life Sciences Imperial College London London UK; ^5^ Shanghai Center for Systems Biomedicine Key Laboratory of Systems Biomedicine (Ministry of Education) Shanghai Jiao Tong University Shanghai China; ^6^ State Key Laboratory for Oncogenes and Related Genes and Bio‐ID Center School of Biomedical Engineering Shanghai Jiao Tong University Shanghai China

**Keywords:** demyelination, Fc monomers, Fc multimers, IgG, immunoglobulin, intravenous immunoglobulin

## Abstract

Intravenous immunoglobulin (IVIG) is an established treatment for numerous autoimmune conditions. Although Fc fragments derived from IVIG have shown efficacy in controlling immune thrombocytopenia in children, the mechanisms of action are unclear and controversial. The aim of this study was to dissect IVIG effector mechanisms using further adapted Fc fragments on demyelination in an *ex vivo* model of the central nervous system–immune interface. Using organotypic cerebellar slice cultures (OSCs) from transgenic mice, we induced extensive immune‐mediated demyelination and oligodendrocyte loss with an antibody specific for myelin oligodendrocyte glycoprotein (MOG) and complement. Protective effects of adapted Fc fragments were assessed by live imaging of green fluorescent protein expression, immunohistochemistry and confocal microscopy. Cysteine‐ and glycan‐adapted Fc fragments protected OSC from demyelination in a dose‐dependent manner where equimolar concentrations of either IVIG or control Fc were ineffective. The protective effects of the adapted Fc fragments are partly attributed to interference with complement‐mediated oligodendroglia damage. Transcriptome analysis ruled out signatures associated with inflammatory or innate immune responses. Taken together, our findings show that recombinant biomimetics can be made that are at least two hundred‐fold more effective than IVIG in controlling demyelination by anti‐MOG antibodies.

## Graphical abstract

Adapted Fc fragments protect against antibody‐mediated demyelination in a dose‐dependent manner where equimolar concentrations of either IVIG or control Fc were ineffective. The protective effects of the adapted Fc fragments are partly attributed to interference with complement‐mediated oligodendroglia damage.

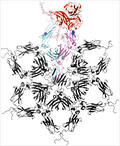

**Abbreviations**
AFM: atomic force microscopy
CNS: central nervous system
Fc: fragment crystallizable
GFP: green fluorescent protein
IVIG: intravenous immunoglobulin

## INTRODUCTION

Clinical trials have established intravenous immunoglobulin (IVIG) as a well‐tolerated, effective drug for the treatment of a wide variety of diseases, ranging from immunodeficiency to autoimmunity [1, 2, 3]. In neurology, high‐dose IVIG (1–2 g/kg) serves as a mainstay therapy in immune‐mediated neuropathies and has been shown to be equally effective as plasmapheresis in Guillain‐Barré syndrome and myasthenic crisis [4]. More recently, IVIG has gained some attention as a treatment option for special forms of inflammatory CNS conditions, which are characterized by pathognomonic autoantibody signatures such as neuromyelitis optica and chronic inflammatory demyelinating polyneuropathy [5].

Mechanistically, many therapeutic modes of action have been attributed to IVIG [6, 7]. While a number of reports described effects of IVIG on various cellular immune components, ranging from T cells [8], B cells [9], NK cells [10] and dendritic cells [11] other authors stress the influence of IVIG on humoral autoimmunity, suggesting anti‐idiotypic antibodies in IVIG [12], FcR engagement [2, 13] and inhibition of complement deposition [14].

This study focused on IVIG effects on antibody‐mediated immune mechanisms. We utilized murine organotypic cerebellar slice cultures (OSCs) as an *ex vivo* model of the immune–CNS interface. As compared to primary cell cultures on the one hand, and animal models on the other, the use of OSC has the advantage that the complex spatial microarchitecture of the CNS is maintained and effector mechanisms of CNS damage can be clearly defined, unobscured by the blood–brain barrier (BBB) and peripheral immune components [15, 16, 17].

Using transgenic mice, which express green fluorescent protein (GFP) in oligodendrocytes and myelin, allowed us to directly monitor demyelination in living OSC. Previously, we had demonstrated that the addition of IVIG efficiently inhibits antibody‐mediated demyelination and microglia activation in OSC of the CNS [18]. This effect clearly depends on the Fc part rather than the antigen‐binding Fab fragment, as IVIG‐derived Fab fragments could neither protect OSC from demyelination nor prevent microglia activation, suggesting a possible direct effect on microglia [18]. While IVIG did not substantially inhibit the binding of the demyelinating antibody to target structures, IVIG‐mediated protection could be prevented with saturating concentrations of complement [18]. This suggests that IVIG may sequester complement making it unavailable to the demyelinating antibody.

The ability of IgG antibodies to bind and activate complement pathways is dependent on both the presence of an attached carbohydrate in the Fc [19] and the ability of IgG to multimerize [20]. Multimerization of injected IVIG may arise through multiple mechanisms that allow a small fraction of injected IVIG to be protective in patients with Guillain–Barré syndrome [20, 21, 22]. These mechanisms can explain why high doses of IVIG are required for protection *in vivo* and why monoclonal IgG (rituximab) is incapable of exerting protection from demyelination [18]

The success of Fc fragments derived from IVIG in treating autoimmune disease in children has motivated the development of recombinant Fc‐based therapeutics [23, 24, 25]. One approach we developed was to create recombinant IgG1‐Fc hexamers by fusing the IgM μ‐tailpiece to the Fc fragment of human IgG1 [24, 26]. The hexamers multimerize by disulphide bond formation at two cysteine residues (Cys‐309 and Cys575) that come from IgM. The molecules also contain an extra N‐linked glycan at Asn‐563 in the IgM μ‐tailpiece. The hexamers are currently in clinical development as they have been shown to block cytotoxicity and pathological changes in experimental in vitro and rat models of neuromyelitis optica through mechanisms that involve interference with complement activation [27, 28].

However, potential drawbacks to the clinical use of hexamers include their large size (~350 kDa), presence of multiple disulphide bonds and a μ‐tailpiece N‐glycosylation site that may all combine to limit manufacture to scale by commercially available cell lines [29]. We therefore created a large panel of cysteine‐ and N‐glycan‐adapted mutants from the parent hexamer [30, 31, 32]. Here, we selected five of these adapted molecules for further study based on their diverse structural and functional properties (Table 1). We show that three of the Fc‐adapted molecules are readily manufactured to scale by CHO‐S cells and are superior to IVIG and wild‐type Fc at controlling demyelination in the OSC model.

**TABLE 1:** Properties of selected leads manufactured in CHO‐S cells

Construct name	IgG1‐Fc	D221N/C309L/N297A/C575A	D221N/C575A	D221N/C309L/N297A/N563A/C575A	D221N/C309L/N563A/C575A	N563A/C575A
Cartoon representation	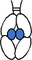	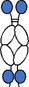	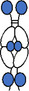	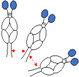	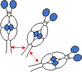	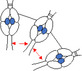
State by SDS‐PAGE	‐monomer	‐monomer	‐monomer ‐dimer ‐trimer Cys‐309 multimers possible	‐non‐covalent monomers	‐non‐covalent monomers	‐covalent multimers Cys‐309 multimers possible
State by SEC	‐monomer	‐monomer	‐monomer ‐dimer ‐trimer	‐non‐covalent multimers	‐non‐covalent multimers	‐covalent multimers
Sialylation	‐simple	‐complex	‐simple	‐complex	‐simple	‐simple
30‐min binding to microglia	+	−/+	++	‐/+	++	+++
24‐h binding to microglia	+	+	++	+	++	+++
Binding to other CNS cells	−	−	−	−	−	−
Binding to low‐affinity Fcγ‐receptors	+	−	+	−/+	++	+++
Binding to glycan receptors	+	+	+	Not tested	++	+++
C1q fixed	‐/+	−	−	−	++	+++
C5b‐9 fixed	−	−	++	−/+	++	−
Inhibits demyelination in OSC model	−	−	++	Not tested	+++	+++

Note

Structures of glycosylated leads as determined by SDS‐PAGE (Figure S1A) and size‐exclusion chromatography (Figure S1B). Blue circles represent location of N‐linked glycans. Glycosylation profiles for each protein were determined by MALDI mass spec (Figure S2). Complex sialylation is defined as the presence of large tri‐ and tetra‐antennary fully sialylated N‐glycans, for example m/z 3776 that are absent from simply sialylated Fcs (Figure S2). Binding of leads to individual human FcγRs, C‐ and I‐type lectins and complement components are shown in Figures S4–S6. Binding to microglia in CNS myelinating cultures is shown in Figure S7.

Abbreviations: SEC, size‐exclusion chromatography; SDS‐PAGE, sodium dodecyl sulphate–polyacrylamide gel electrophoresis; red arrows indicate the 18 amino acid tailpiece that contains hydrophobic amino acids that encourage non‐covalent attraction of Fc monomer units.

## MATERIALS AND METHODS

### Generation of de‐glycosylated IVIG

To avoid endotoxin contamination, PNGase F was expressed in ClearColi® BL21(DE3)‐electrocompetent cells (Lucigen). Shortly, ClearColi cells were electroporated with PNGase F plasmid pOPH6 [33] (kindly provided by Prof. Max Crispin, University of Southampton, UK) and expanded in LB‐Miller medium (Sigma‐Aldrich, Hamburg, Germany) with Ampicillin (final concentration 100 µg/ml; Sigma‐Aldrich, Hamburg, Germany). PNGase F expression was induced by addition of Isopropyl β‐D‐1‐thiogalactopyranoside (final concentration 1 mM) at OD_600_ = 0·6. Harvested bacteria underwent enzymatic and mechanical lysis and were purified using His GraviTrap™ columns (GE Healthcare) according to the manufacturer's instructions. For de‐glycosylation under non‐denaturing conditions, 15 mg of IVIG (Privigen®, CSL Behring) was incubated with 60 µg of PNGase F in 1x GlycoBuffer (NEB) at 37°C for 24 h. De‐glycosylated IVIG was purified over a protein G column (GE Healthcare), dialysed against PBS and concentrated using Amicon® Ultra‐15 Centrifugal Filter Units (Merck Millipore).

### Generation of Fc fragments

Adapted Fc constructs manufactured in CHO‐K1 or HEK293‐F cells have previously been described [30, 31, 32]. To generate the quantities of Fc fragments required for this study, manufacture of selected leads (defined in Table 1) was outsourced to Abzena Ltd for synthesis in their suspension‐adapted CHO‐K1 cells (originally received from ATCC and adapted to serum‐free growth in suspension culture). Constructs were gene‐synthesized and cloned into the proprietary expression vector, pEVI‐5, using conventional (non‐PCR based) methods. Plasmid DNA was prepared, cells were transfected with eviFect, a proprietary transfection reagent, and cells were grown after transfection in eviMake, an animal component‐free, serum‐free medium. Supernatants were harvested by centrifugation and subsequent filtration (0·2‐μm filter).

Fc fragments were purified from cell culture supernatant using a 5 ml HiTrap Protein G HP column (GE Healthcare). The supernatants for each mutant were processed as two separate batches. Each time, the column was washed using 20 mM sodium phosphate pH 7·2 containing 150 mM NaCl and protein eluted using 0·1 M glycine pH 2·7. Collected fractions were neutralized using 1 M Tris pH 9·0 and then buffer‐exchanged using Zeba™ Spin 7 K MWCO, 10 ml Desalting Columns (Thermo Fisher, Loughborough, UK), into 10 mM sodium acetate pH 5·6 buffer containing 100 mM NaCl. The final samples were then filter‐sterilized before quantification by A280 nm using an extinction coefficient Ec (0·1%) based on the predicted amino acid sequence. Purified proteins were then analysed by SDS‐PAGE (Figure S1A) and analytical SEC‐HPLC (Figure S1B). All the glycan‐adapted Fcs, with the exception of the D221N/C309L/N297A/N563A/C575A mutant, could be made by Abzena to the g/L yields required for translating to *in vivo* studies. Consequently, we were unable to test the D221N/C309L/N297A/N563A/C575A mutant in the OSC model.

### Animals

C57BL/6 mice were used to generate CNS myelinating cultures for binding studies as described previously [34]. All animal studies were approved by the Ethical Committee of the University of Glasgow and licensed by the UK Home Office [34, 35, 36]. For the OSC experiments, mice were bred at the animal facility of the Heinrich Heine University Duesseldorf under specific pathogen‐free conditions. Dr B. Zalc kindly provided PLP‐GFP mice in which GFP is expressed under regulatory elements of the PLP gene in oligodendrocytes and located in the cytosol and in the myelin sheath (B. Zalc, personal communication) [37, 38]. Dr. M. Gliem (Heinrich‐Heine, Duesseldorf) kindly provided CX3CR1 mice (B6·129P‐Cx3cr1^tm1Litt^/J) in which the gene of the chemokine receptor CX3CR1 is replaced by GFP. In the CNS, CX3CR1 is dominantly expressed in microglia [39]. All the animal experiments described comply with the laws of both the UK and Germany.

### Organotypic cerebellar slice culture

Organotypic cerebellar slice cultures were prepared according to a modified protocol published by Stoppini et al.[40] Shortly, P9‐P10 pups were anaesthetized, and killed, and the cerebellum was removed and cut into 400‐μm‐thick slices using a McIlwain tissue chopper. OSCs were then dissected manually in ice‐cold dissecting medium and transferred to washing medium for 10 min on ice. Thereafter, OSCs were cultured on cell culture inserts with a pore diameter of 0·4 µm (Millipore) in culture medium at 37°C and 5% CO_2_ for 3–5 days and subsequently at 33°C and 5% CO2 for the duration of the experiment. The different media used were as follows: dissecting medium—Hank's Balanced Salt Solution with calcium and magnesium (HBSS), 100 U/ml penicillin, 100 μg/ml streptomycin (all from Thermo Fisher Scientific), 5 mg/ml glucose and 1 mM kynurenic acid (both from Sigma‐Aldrich, Seelze, Germany), pH 7·2‐7·4; washing medium—50% HBSS and 50% Minimum Essential Medium (MEM), supplemented with P/S and 25 mM HEPES (all from Thermo Fisher Scientific); and culture medium—50% MEM supplemented with P/S, 25% HBSS supplemented with P/S, 25% horse serum (Thermo Fisher Scientific), 5 mg/ml glucose and 2 mM glutamine.

### Demyelination

For demyelination experiments, culture medium was supplemented with 5 µg/ml recombinant humanized 8‐18c5 (hu8‐18c5 IgG1 kappa directed against MOG [15, 41] and 6‐8% pooled normal baby rabbit serum [BRS]). Therefore, lyophilized BRS reconstituted in 1 ml of water was used as a source of complement (Cedarlane). For clarity, BRS is referred to as ‘complement’. Alternatively, 11% human serum (Sigma‐Aldrich) was used as a source of complement where indicated. IVIG (Privigen®; 0·9 µg/ml–6 mg/ml) or variably glycosylated recombinant human IgG1‐Fc fragments (0·3 µg/ml–1 mg/ml) were added during demyelination as indicated. Prior to use, IVIG was dialysed against HBSS.

### Quantification of fluorescence in OSC

For the quantification of the relative myelin content of OSC during demyelination, we assessed the intensity of GFP expression in living OSC at different time points during each experiment: using ImageJ software, we assessed the area of fluorescence signal exceeding a defined threshold in digital images acquired with an Olympus BX51 microscope at 4x magnification. The threshold was chosen according to the background intensity, and only the specific GFP expression was quantified. For each individual OSC, results are depicted relative to time point 0, prior to the beginning of the experiment.

### Immunostaining of OSC

Organotypic cerebellar slice cultures were fixed in 4% formaldehyde (Carl Roth), permeabilized with 1% Triton X‐100 (Thermo Scientific) in PBS for 30‐45 min and blocked with 10% normal goat serum (Sigma‐Aldrich) in 0·2% Triton X‐100 in PBS for 1 h after three washes with PBS. Primary antibodies (anti‐Iba1 Wako, Neuss, Germany; anti‐CD68, Biolegend) were diluted in PBS supplemented with 1% goat serum and 0·2% Triton X‐100, according to the manufacturer's instructions, and were incubated for 1–2 days at 4°C. After three consecutive washes in PBS, OSCs were incubated overnight with secondary antibodies (goat α rabbit/rat‐Cy3, Millipore), diluted in PBS, supplemented with 1% goat serum and 0–2% Triton X‐100 according to the manufacturer's instructions at 4°C in the dark. Images were acquired using a Leica SP8 confocal laser‐scanning microscope (Wetzlar) and analysed with LAS X software (Leica) and ImageJ. Some images were contrast‐enhanced to facilitate visibility in composite figures.

### Immunostaining of CNS cultures

For immunostaining, CNS myelinating cultures were treated with Fc fragments (20 µg/ml) for 30 min at 4°C or 24 h at 37°C and then fixed with 4% paraformaldehyde for 20 min at room temperature and subsequently stored in PBS at 4°C. Three coverslips were used for each condition and N. Post‐fixation, cultures were permeabilized for intracellular staining in Triton X‐100 (0·5%; 10 min), washed and non‐specific binding blocked with 1% BSA/ 10% horse serum in PBS. Primary antibodies were applied in blocking buffer for 45 min at room temperature. To label microglia, rabbit anti‐Iba1 (Wako/ Alpha laboratories; Richmond, VA, USA/ Eastleigh, Hampshire, UK; 1 in 500) was used. To label axons together with myelin, rat anti‐MBP (myelin basic protein) (AbD Serotec, Kidlington, UK; 1 in 500) was used in combination with mouse SMI31 anti‐phosphorylated heavy‐ and medium‐chain neurofilament (Biolegend; 1 in 1500). After washing, secondary antibodies (goat anti‐human IgG (Fcγ fragment specific), goat anti‐mouse IgG1 or goat anti‐rabbit IgG or goat anti‐rat IgG; all 1 in 400; Jackson ImmunoResearch, Cambridgeshire, UK, and Thermo Fisher Scientific, Waltham, MA, USA) were applied for 15 min at room temperature. Coverslips were mounted on glass slides in either CitiFluor mounting medium with DAPI (1 ng/ml; Electron Microscopy Sciences) and sealed with nail enamel or Mowiol mounting medium (4·2% glycerol [w/v], 0·4% Mowiol 4‐88 [w/v] (Calbiochem), 2·1% 0·2 M Tris pH 8·5 [v/v]) with DAPI (1 ng/ml).

### Image capture and analysis of CNS myelinating cultures

Representative images and images for quantification were captured by using an Olympus BX51 fluorescence microscope and Ocular software (QImaging, Teledyne Photometrics). To avoid any bias, areas (field of view) for quantification were selected in the DAPI channel (blue). Images in all three channels were then captured (blue/red/green). For quantification, 10 images were captured per coverslip using x10 for quantification of myelin and axons.

Myelin (MBP) and axons (SMI31‐neurofilament) were quantified by using the CellProfiler software, and the pipelines are available at https://github.com/muecs/cp.[42]

### RNA analysis

Mouse CNS myelinating cultures were incubated with Fc fragments at 20 µg/ml or in media alone for 24 h. After removal of culture media, cells were lysed in TRIzol (Thermo Fisher Scientific) for 10 min at room temperature; lysate was stored at −80°C until required. For RNA sequencing, we purified the RNA from 6 coverslips per N for each treatment group (*N* = 4) using PureLink^TM^ RNA Mini Kit (Thermo Fisher Scientific) according to the manufacturer's instructions with an on‐column DNA digestion step (PureLink^TM^ DNase set, Thermo Fisher Scientific). The RNA concentrations were measured with a Qubit Fluorometer (Thermo Fisher Scientific), and the RNA integrity was determined using an Agilent 4200 TapeStation (Agilent Technologies). Samples had an average RIN of 9·3. 500 ng of total RNA from each sample was used to prepare libraries for sequencing, using an Illumina TruSeq Stranded mRNA HT Kit (Illumina), according to the manufacturer's instructions. Briefly, polyadenylated RNA molecules were captured, followed by fragmentation. RNA fragments were reverse‐transcribed and converted to dsDNA, end‐repaired, A‐tailed, ligated to indexed adaptors and PCR‐amplified. Libraries were pooled in equimolar concentrations and sequenced in two high output cartridges, on an Illumina NextSeq 500 sequencer (Illumina) using a high output cartridge, generating approximately 28·5 million single reads per sample, with a length of 75 bp. At least 93·9% of the reads generated, presented a Q score of 30 or above. Prior to performing bioinformatics analysis, RNA‐seq read quality was assessed using FastQC software (http://www.bioinformatics.babraham.ac.uk/projects/fastqc). Sequence adaptors were removed using TrimGalore (https://www.bioinformatics.babraham.ac.uk/projects/trim_galore/), and RNA‐seq reads were analysed. Sequence reads were aligned to the *Mus musculus* genome (GRCm38), downloaded via Ensembl using HISAT2. HISAT2 is a fast and sensitive splice‐aware mapper, which aligns RNA sequencing reads to mammalian‐sized genomes using FM index strategy [43]. After the alignment, FeatureCounts [44] was used to count the reads mapping to the reference genome. The edgeR package was used to calculate the gene expression level and to analyse differentially expressed genes [45]. For cut‐off, P‐value <0·05 was used in edgeR.

The raw RNA sequencing data were submitted to the European nucleotide archive under accession number PRJEB41654.

### AFM imaging

The stock solution of D221N/N563A/C575A was diluted to 10 μg/ml in 0·2X HBSS buffer and then directly applied to a freshly cleaved fragment of muscovite mica. After incubating for 10 min, the sample was rinsed extensively with 0·2X HBSS buffer to remove loosely adsorbed molecules. The sample was then fixed with 2% glutaraldehyde for 5 min, followed by extensive washing with 0·2X HBSS buffer and quenching of the fixation with 20 mM glycine. Imaging was performed with the PeakForce tapping mode with a Multimode AFM (Bruker, Santa Barbara, CA) using Scanasyst‐Fluid+cantilevers (Bruker) with a spring constant of 0·7N/m in 0·5x HBSS buffer. For all images, the scan rate was 1·51 Hz with a frame size of 384 x 384 pixels, using a PeakForce frequency of 2 kHz and an oscillation amplitude of 30 nm. The force set‐point was 100 pN, and an automatic gain control was used to minimize the PeakForce error. Image analysis was performed using NanoScope analysis v1·80 (Bruker Nano Surfaces, Santa Barbara). The lateral dimensions of individual domains were determined from the full width at half height, and the inter‐domain distances were measured from the peak heights of each domain. It should be noted that we examined several mutants with AFM and all showed the general features (at larger scan sizes) as described here for D221N/N563A/C575A. However, we found that only the D221N/N563A/C575A molecules were sufficiently strongly adsorbed onto mica to be able to resolve the finer features.

### Statistics

Analyses were performed by using GraphPad Prism 8 software (GraphPad software Inc.). A paired, two‐tailed Student's *t* test was used to compare two groups with significance (*p*‐value) indicated or as described in the accompanying legends.

## RESULTS

### Removal of N‐linked glycans from ivig strongly reduces inhibition of immune‐mediated demyelination

**FIGURE 1:**
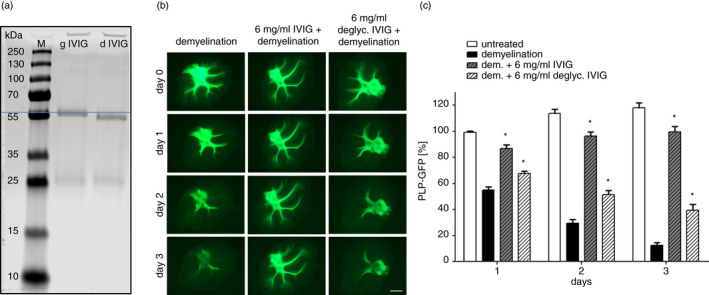
Therapeutic properties of IVIG depend, at least in part, on glycosylation. IVIG was de‐glycosylated, under non‐denaturing reaction conditions by recombinant PNGase F. De‐glycosylated IVIG and glycosylated (‘wild‐type’) IVIG were used to treat OSC during immune‐mediated demyelination. (a) SDS‐PAGE analysis of de‐glycosylated (dIVIG) and glycosylated IVIg (gIVIG) under reducing and denaturing conditions: The shorter molecular size of dIVIG supports successful de‐glycosylation. (b) De‐glycosylated IVIG has reduced protective properties as compared with wild‐type IVIG: OSC was demyelinated with 5 µg/ml anti‐MOG antibody and 8% complement (left column) in the presence or absence of either glycosylated or de‐glycosylated IVIG (6 mg/ml). Demyelination was visualized and quantified via GFP expression of oligodendrocytes and myelin under regulatory elements of the myelin protein PLP. For the quantification of the relative myelin contents of OSC during demyelination, PLP‐GFP expression was documented daily in living OSC: While glycosylated IVIG protect from immune‐mediated demyelination (middle column), this protection was reduced when using de‐glycosylated IVIG (right column), scale bar =1 mm. (c) Quantification of the GFP+area relative to day 0 (*n* = 7–8 OSC per group); PLP‐GFP expression confirmed reduced protection by de‐glycosylated IVIG. Significant differences were calculated with respect to the demyelinated control using one‐way analysis of variance and Dunnett's post hoc test. *p ≤ 0·05. Values are depicted as mean ± SEM. GFP, green fluorescent protein; IVIG, intravenous immunoglobulin; OSC, organotypic cerebellar slice culture

In therapeutic approaches in which the Fc of human IgG1 is critically important, receptor binding and functional properties of the Fc, including C1q binding, are lost after de‐glycosylation or removal of the Asn‐297N‐linked attachment site [13, 19, 46]. To determine whether N‐linked glycosylation is critical to the ability of IVIG to inhibit immune‐mediated demyelination, we used an excess of PNGase F to remove the sugars from IVIG (Figure 1a). We next induced demyelination in OSC with 5 μg/ml recombinant humanized MOG‐specific antibody and 6–8% complement as previously described [15]. This treatment induces rapid and pronounced demyelination, which can be monitored by quantification of the GFP fluorescence emitted by intact myelin/oligodendrocytes (as described in Materials and Methods) before and during treatment. Whereas the addition of >6 mg/ml IVIG significantly reduced the decrease in GFP fluorescence in OSC treated with anti‐MOG antibody and complement, equivalent concentrations of the de‐glycosylated IVIG were significantly reduced in protecting from demyelination (Figure 1b,c). Neither fully glycosylated, nor de‐glycosylated IVIG, in the presence of complement had any effects on myelin integrity in the absence of the anti‐MOG antibody. Taken together, these data show that N‐linked glycans attached to the Fc are important for the observed inhibition of demyelination by the Fc.

### Glycosylation‐adapted recombinant FCS inhibit immune‐mediated demyelination in a dose‐dependent manner

The observation that the presence of N‐linked glycans on IVIG was essential to its mechanism of action in the OSC model led us to further explore the role of N‐linked glycosylation using recombinant Fcs into which enhanced N‐linked glycosylation was engineered [30, 31, 32]. From our previous work, we shortlisted five combinations of glycosylation and cysteine substitution mutants, which formed either monomers or multimers and possessed different binding characteristics for FcγR, C‐ and I‐type lectins and complement components (see Table 1 and Figures S1‐S6 for binding experiments). As sialylation of IgG‐Fc domains is believed to also be important for the anti‐inflammatory effects of IVIG [47], molecules containing simple mono‐antennary sialylation and larger more complex tri‐ and tetra‐antennary sialylation were selected for study (Figure S2).

**FIGURE 2:**
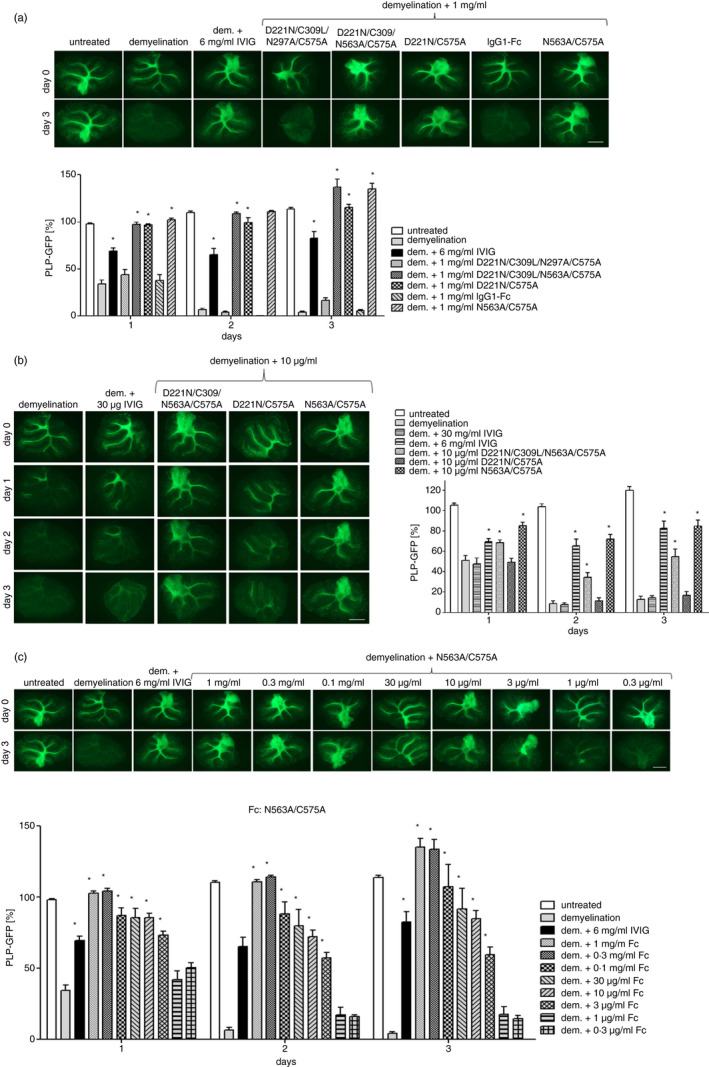
Glycosylated Fc leads show variable protection from immune‐mediated demyelination. (a) OSCs were demyelinated with 5 µg/mL anti‐MOG antibody and complement for three days absence (column 1) or presence of either WT‐IVIG (6 mg/ml, column 3); non‐protecting Fc backbone (1 mg/ml, column 7) or different glycosylated Fc leads (protective Fc constructs in columns 5, 6, 8; 1 mg/ml); n = 4‐20 OSC per group) (b) Two protective glycosylated Fc leads (D221N/C309L/N563A/C575A and N563A/C575A) inhibit demyelination at 10 µg/ml (*n* = 12–17 OSC per group). (c) Titration of the N563A/C575A mutant shown in (a) and (b). Demyelination was quantified by GFP fluorescence emitted from myelin of OSCs prepared from transgenic mice expressing GFP under regulatory elements of the myelin protein PLP (*n* = 4–20 OSC per group). Scale bars 1 mm. Significant differences were calculated with respect to the demyelinated control using one‐way analysis of variance and Dunnett's post hoc test. *p ≤ 0·05. Values are depicted as mean ± SEM. GFP, green fluorescent protein; IVIG, intravenous immunoglobulin; OSC, organotypic cerebellar slice culture

We found that, whereas axons in the anti‐MOG/complement‐treated group were demyelinated, myelin was preserved significantly with as little as 10 µg/ml of D221N/C575A, 10 µg/ml D221N/C309L/N563A/C575A or 3 µg/ml of N563A/C575A (Figure 2, Figure S3). Concentrations of IVIG, recombinant IgG1‐Fc or D221N/C309L/N297A/C575A to 1 mg/ml were ineffective (Figure 2a). Only at very high concentrations of IVIG (6 mg/ml) could myelin integrity be preserved in the OSC model (Figure 2). The D221N/C575A, D221N/C309L/N563A/C575A and N563A/C575A molecules also protected myelin in OSCs when demyelination was induced with human instead of rabbit complement (Figure S3). All the Fc molecules that so effectively controlled demyelination in the OSC model also fixed complement more efficiently (Figures S3 and S4), and bound low‐affinity FcγRs (Figure S5) and glycan receptors (Figure S6) when compared to control molecules and as described previously [30, 31, 32] (summarized in Table 1). Assessing the binding to cells in mouse CNS myelinating cultures showed notably enhanced binding to, and uptake by microglia, for the N563A/C575A and D221N/C309L/N563A/C575A molecules (Figure S7). Despite binding and uptake into microglia, none of the glycan‐adapted Fc molecules negatively impacted the viability of axons or myelin as assessed by immunofluorescence microscopy (Figure S7D,E).

### Glycosylation‐adapted recombinant FCS do not trigger inflammatory signatures from myelinating CNS cultures as revealed by transcriptome analysis

To determine whether Fc binding and uptake into microglia (Figure S7) can trigger inflammatory signatures, we performed transcriptome analysis of total RNA from myelinating CNS cultures treated with the different glycan‐adapted Fc molecules for 24 h. Two analyses were undertaken: one comparing relative transcript abundance of each Fc against the media control (Figure S9), and a second analysis comparing transcript abundance against the IgG1‐Fc control (Figure S10). Despite the close structural relatedness of these molecules, most differentially expressed genes were unique to each construct in both analyses (Figure S9 and S10), with only three shared downregulated genes (*mt*‐*Nd6*, *Gm28437* and *Hnrnpu*) seen in all the treatment groups (Figure S9). Many processed pseudogenes were observed in the transcriptomes, including *Gm8325*, *Hnrnpu*, *H19* and *Malat*‐*1*. Some of these are protein coding with unknown function, while others can be processed into short interfering RNAs (siRNAs) or long intergenic non‐coding RNAs (lincRNA) that can potently regulate coding genes [48]. Gene ontology analysis revealed very few immune‐ and inflammatory response‐associated genes in contrast to transcriptomic studies of mouse CNS after viral infection where multiple immune genes are transcribed [49]. One notable gene whose transcript number decreased significantly in response to all the Fc treatments was *Hnrnpu* (Figure S9). Also known as nuclear matrix protein SAFA, *Hnrnpu* functions as a nuclear viral dsRNA sensor for both DNA and RNA viruses [50]. Upon recognition of viral dsRNA, SAFA oligomerizes and activates the enhancers of antiviral genes, including IFNB1 whose transcripts are notably absent in our analysis. Differentially expressed genes (DEG) were analysed using P‐values as shown in Figures S9 and S10, and it is important to add that the more stringent analysis by *Q* value <0·05 did not show differences between treatment groups. The relatively small number of DEGs identified by P‐value may therefore need further investigation to assess the effects, and it cannot be excluded that small differences are due to the biological replicates. These results now form the basis for further microglia‐specific analyses into the effects of the Fc in the presence of the anti‐MOG antibody and complement.

### Reduction in CD68 and Iba1 expression on glial cells by the adapted N563A/C575A Fc

**FIGURE 3:**
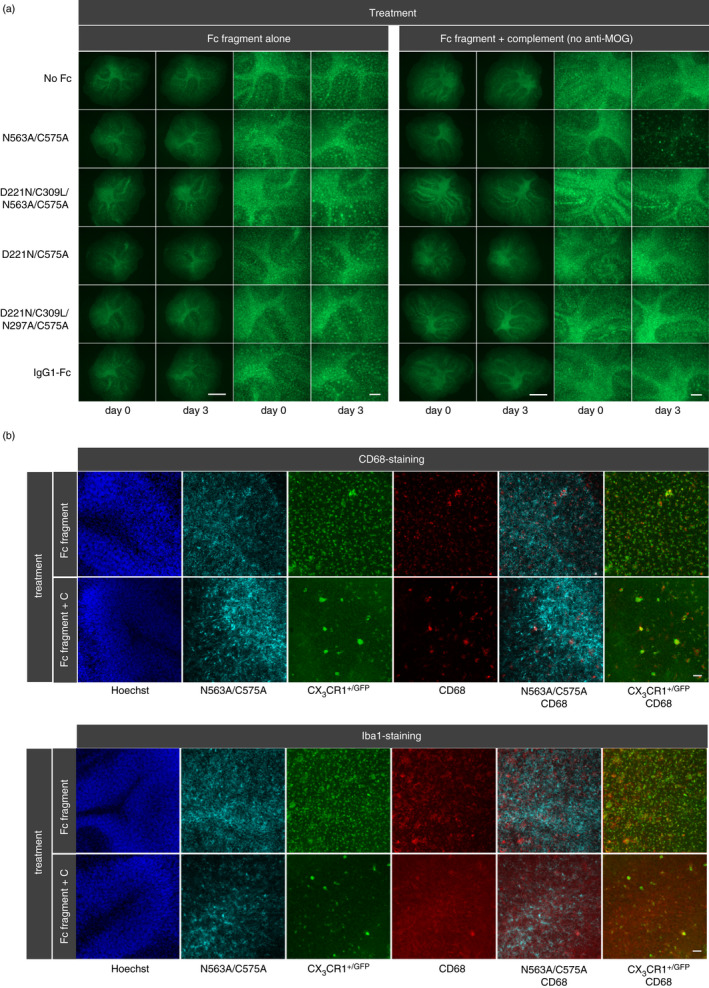
Incubation of OSCs from CX_3_CR1^+/GFP^ transgenic mice with Fc fragments. OSCs were incubated with 10 µg/ml fluorescence‐labelled recombinant Fc fragments either alone or together with 7% rabbit complement, and in the absence of the anti‐MOG antibody. (a) CX_3_CR1^+/GFP^‐positive microglia cells were reduced by treatment with N563A/C575A and complement but not by treatment with N563A/C575A alone. The other Fc constructs did not affect CX_3_CR1^+/GFP^‐positive microglia cells. Overviews: scale bar 1 mm, enlargement: scale bar 250 µm. (b) After three days, OSCs were fixed, stained with either anti‐Iba1 antibody or anti‐CD68 antibody to analyse microglia and activated microglia via confocal microscopy. In OSCs treated with N563A/C575A and complement, signals of Iba1 and CD68 were reduced. Stack images were taken with a distance of 5 µm and displayed as maximum projection (scale bar = 50 µm). Similar results were observed in experiments where the anti‐MOG antibody was included with the complement (Table S1). OSC, organotypic cerebellar slice culture; MOG, myelin oligodendrocyte glycoprotein

Microglia are an important population of Fcγ‐receptor bearing phagocytic cells in OSC whose activation and/or inhibition can be modulated by IVIG through different mechanisms that involve both Fc‐ and Fab‐dependent pathways [51, 52, 53, 54]. To determine what impact the glycosylation‐adapted Fcs have on microglia during rescue from demyelination induced by the anti‐MOG antibody and complement, we used OSCs from CX3CR1‐/GFP heterozygous transgenic mice, whose microglia express both GFP and CX3CR1 [39]. All the molecules with the exception of the D221N/C309L/N297A/N563A/C575A mutant clearly bound microglia with no typical axonal or myelin binding pattern seen (Figure S7). Three Fc molecules (D221N/C575A, D221N/C309L/N563A/C575A and N563A/C575A) interacted particularly strongly with microglia as binding was also seen after shorter incubations of 30 min at 4°C (Figure S7A). We observed pronounced loss of CD68 and Iba1 staining of microglia with as little as 10 μg/ml of the N563A/C575A molecule when incubated with 7% complement, in the presence or absence of the added anti‐MOG IgG that was not observed with the other Fc molecules (Figure 3 and Table S1).

### Structures of N563A/C575A tailpieces reveal potential mechanism for C1q binding

As discussed above, molecules in which both the tailpiece‐attached glycans (Asn‐563) and cysteine (Cys‐575) are removed have a propensity to multimerize in the absence of any associating proteins such as J‐chain or secretory component, thus facilitating interactions with complement component C1q. Recent cryo‐EM structures of IgA [55] and IgM [56] have shown that the tailpieces form β‐strands that pack into an amyloid‐like β‐sandwich in which Tyr‐562, Val‐564, Leu‐566 and Met‐568 mediate hydrophobic interactions to stabilize the multimer. In the absence of tailpiece glycans, tailpiece cysteines or tailpiece‐associating proteins, β‐strand formation may be a sufficient driving mechanism for multimerization of the Fcs to occur. To determine whether this is the case, we imaged a tailpiece‐adapted molecule D221N/N563A/C575A by PeakForce AFM under solution.

**FIGURE 4:**
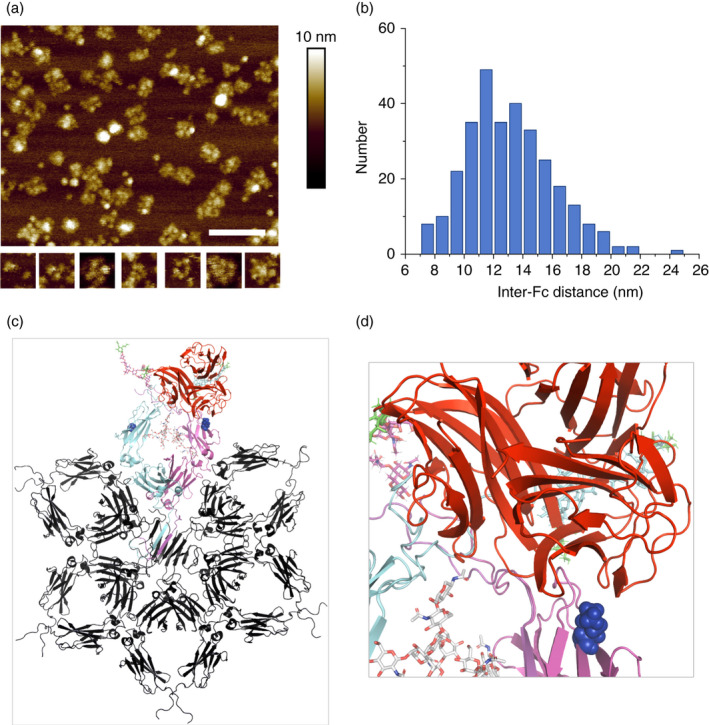
A model for Fc multimerization and interaction with C1q. (a) AFM images of the D221N/N563A/C575A molecule reveal a wide range of structures and sizes, many of which clearly consist of multiples of globular elliptical domains that are of the expected size (~10 to 12 nm in lateral dimension, ~3 nm in height) of individual Fc fragments. The lower panels (each 38 × 38 nm^2^) show isolated complexes containing from one to more than eight Fc fragments. Scale bar: 100 nm. (b) Distribution of the distances between individual Fc fragments within single complexes, showing that most of these distances are within the range of inter‐C1q head distances (9 to 18 nm) observed in C1q/IgG and C1q/IgM complexes. (c) The D221N/N563A/C575A aligned in PyMOL onto the cryo‐EM structure of IgM (pdb; 6KXS, shown in black). One Fc heavy chain is shown in cyan, the other in magenta, and the N‐linked oligosaccharides attached to the hinge are shown in their respective heavy‐chain colour terminating in sialic acid (green). The 7Å structure of C1q in complex with IgG1 (pdb, 6FCZ) allowed the globular head domains of C1q (shown in red) to be mapped onto the Fc. Residue Lys‐322 that is critical for C1q binding is shown in blue spheres on each of the Fc heavy chains. (d) Panel C magnified to show the accessibility of the C1q binding site

We observed a wide range of structures (Figure 4a). Each individual structure consisted of a number (from 1 to 11, mode 6, *n* = 36) of globular elliptical domains of lateral dimension, 9·5 ± 1·5 nm by 12·4 ± 1·8 nm, and height, 3·1 ± 0·7 nm (*n* = 31), consistent with the size of an individual Fc fragment (with AFM tip broadening). While these structures were highly variable, there was nonetheless a common feature: other than the single‐domain structures, the domains were radially disposed around a central location (see the isolated panels in Figure 4a), resembling a star‐shaped structure. Such an architecture is consistent with a central β‐sheet of the tailpieces with a radial disposition of the Fc domains. We speculate that the variability in stoichiometry is owing to the amyloid‐like β‐sandwich, which can indeed accommodate a range of stoichiometries in IgA and IgM [41, 42], while the variability in structure is a result of the absence of any other favourable inter‐domain interactions other than that mediated by the tailpieces.

Structural studies of C1q bound to hexameric IgG or IgM have shown that the C1q binding sites in these antibody complexes are radially arranged, with binding sites spaced between ~9 nm and ~18 nm from each other [57]. Further, this work has also shown that there is some flexibility in the C1q structure bound to these complexes, with the maximal distance of the C1q head domains ranging from 19 nm to 24 nm. Thus, we also examined the inter‐domain distances of the Fc‐complexes in these AFM images, finding that most domains are between 7 nm and 22 nm apart from each other (Figure 4b), which is within the same range as the inter C1q‐binding site distances in IgG and IgM. Thus, as C1q binding only appears to require a radial arrangement of binding sites within this distance range, our images suggest that these complexes might indeed bind C1q in an overall roughly similar manner as with IgG and IgM, except that many different multivalent binding architectures are possible with these highly variable Fc‐complexes.

## DISCUSSION

In this study, we aimed to assess direct protective effects of variably glycosylated Fc fragments on CNS tissue during antibody‐mediated demyelination [58]. To mimic immune‐mediated demyelination, OSCs were incubated with a humanized recombinant anti‐MOG antibody together with complement for 3 days. At the concentrations of anti‐MOG antibody and complement tested, this treatment resulted in rapid and severe demyelination and damage of oligodendrocytes, while axons remained largely intact for the duration of the experiment as shown before [18]. The effect of IVIG, but not de‐glycosylated IVIG (Figure 1), in blocking this immune‐mediated demyelination argues for a mechanism of action that is dependent on IgG glycosylation, but that does not require sialic acid or variable region binding through the Fab antigen binding sites. IVIG clearly reduced demyelination of axons as depicted in Figure 1b, while equimolar concentrations of de‐glycosylated IVIG or Fab fragments as previously published [18] had little or no effect.

These findings led us to investigate the efficacy of a panel of glycan‐modified human IgG1‐Fcs engineered (Figure 2 and Table 1) for enhanced glycosylation [26, 27, 28] that allow for high avidity binding to low‐affinity receptors (Figures S5 and S6). Because complement activation by IgG is known to be critically dependent on Fc glycosylation and multimerization [19, 20], and because we had previously shown that protective effects of IVIG were reliant on the presence of complement [18], we selected Fc molecules with a variety of complement fixation profiles (Figure S4). We designed molecules that could fix complement either through the classical (as with D221N/C309L/N563A/C575A) or lectin (as with D221N/C575A) pathway and could show that the molecules were at least 50‐ and 20‐fold, respectively, more effective at inhibiting demyelination by the anti‐MOG antibody than IVIG at 6 mg/ml (Figure 2) or Fc fragments at 2 mg/ml derived from IVIG seen previously [18]. Although we do not yet fully understand the reasons for their efficacy, we propose that one mechanism involves sequestering complement components making them unavailable to the demyelinating anti‐MOG antibody, and as previously shown with hinge‐adapted Fc multimers [59].

No significant effect on the viability of microglia or myelinating CNS cultures was observed when incubated with any of the Fc molecules alone (Figure S7). We could show that the same Fc molecules that inhibit demyelination were also capable of binding Iba+microglia cells in mouse myelinating CNS cultures (Figure S7). The binding to microglia cells clearly does not directly impact on the ability of these human Fcs to inhibit complement‐mediated anti‐MOG demyelination in the OSC model, and the transcriptomic data derived from myelinating CNS cultures treated with the Fc molecules alone did not show increased gene signatures for microglia activation (Figures S9 and S10). Intriguingly, significant deregulation of both *Hnrnpu* [60] and lincRNAs [61] has previously been reported from peripheral blood taken from chronic inflammatory demyelinating polyradiculoneuropathy patients, although our report is the first investigation of the direct impact of Fc fragments on myelinating CNS cultures.

In the absence or presence of the demyelinating anti‐MOG antibody, we observed significant loss of CD68‐ and Iba1‐positive microglia when OSCs are incubated with complement and the N563A/C575A Fc (Figure 3). Although we do not know the mechanism behind the reduced GFP/CD68/Iba1 expression by the N563A/C575A Fc, removal of microglia by clodronate treatment also prevents demyelination by the anti‐MOG antibody, showing that microglia must also be involved (Figure S8). Although we currently do not know whether the inhibition of demyelination by the N563A/C575A Fc is also a consequence of reduced GFP+microglia, studies by other groups have shown that temporary microglial depletion followed by their repopulation from the periphery can lead to a broad range of positive and neuroprotective outcomes in distinct disease conditions by reducing neuroinflammation [62].

Many studies have shown anti‐inflammatory and tolerogenic properties of IgG‐Fc aggregates at suppressing ongoing autoimmunity in a variety of animal models of neurological disease [59, 63, 64]. Several autoantibodies against complement factors have been described, and in other studies, anti‐idiotypic effects of IVIG were found responsible for scavenging complement in various model systems [59, 65, 66, 67]. We clearly show that blocking of complement by IVIG does not involve specific antigen binding via the variable domains (Fab fragments) as the recombinant Fc molecules tested do not possess such domains. Instead, our findings confirm that the Fc is sufficient to interfere with the complement cascade, in line with Piepers *et al*,[14] although we do not yet know the exact mechanism of this interference. Non‐covalent interactions between Fc segments of IgG have been shown to result in the formation of ordered IgG hexamers after antigen binding on cells [20, 68, 69]. These IgG hexamers recruited and activated the complement cascade and could be further engineered into therapeutic IgGs for enhancement of complement activation and killing of target cells.[20] Direct imaging and modelling of N563A/C575A containing Fc multimers likewise offer a structural explanation for how the globular head domains of C1q can readily access their binding site at the top of the Fc Cγ2 domains (Figure 4), and thereby making C1q unavailable to the demyelinating anti‐MOG antibody.

Both animal experiments and clinical evidence suggest that the modification pattern of the Asn‐297‐attached Fc carbohydrate is crucial for the Fc‐mediated therapeutic effects of IVIG. Ravetch and colleagues showed that sialic acid residues attached to the IgG Fc region seem to be indispensable for certain effects [70, 71]. Sialylated IgG preferably interacts with C‐type lectins such as human DC‐SIGN (Dendritic Cell‐Specific Intercellular adhesion molecule‐3‐Grabbing Non‐integrin) or the murine homolog SIGNR1 [72]. Park *et al*. demonstrated murine SIGNR1 expressed on microglial cells in the cerebellum, identifying microglia as a likely target for IVIG interference [73]. However, the relative abundance of complex sialylated structures on the D221N/C309L/N297A/C575A mutant that did not protect, against the paucity of sialylated structures on the N563A/C575A mutant that did protect in the OSC model, argues against a direct role for sialic acid (Figure S2). This observation is supported by studies in a number of autoimmune disease models that have shown the protective effects of IVIG to be largely independent of sialylation or interactions with DC‐SIGN [74, 75]. However, the sialylation state of the Fc may become more critical in the presence of a BBB, as influx and efflux of IgG in both human and mouse studies have been shown to be glycan‐ and sialic acid‐dependent [76, 77, 78, 79, 80]. Importantly, transcriptome analysis shows that there is no induction of inflammatory or innate immune responses, such as those, for example, with virus infection.

We therefore propose that in the complex microenvironment of the CNS, Fc‐mediated effector mechanisms of IVIG may play a pivotal role, possibly acting on both microglia and complement factors in parallel by distinct or cumulative mechanisms. In summary, we demonstrate that the Fc modulates immune–CNS interactions in a multimodal fashion. In this model of immune (antibody/complement)‐mediated demyelination, the preservation of myelin seems to largely depend on the inhibition of the complement cascade by the Fc. Additionally, Fc‐mediated alterations to microglia expression of CD68/Iba1 may be an important effect of IVIG and these glycan‐adapted Fcs even though the mechanisms have yet to be determined. Local apoptotic‐like mechanisms are known to underlie complement‐mediated synaptic pruning by microglia [81], and it may be that similar mechanisms in the presence of the glycan‐adapted Fcs (e.g. N563A/C575A) can control microglia functionality with respect to preventing demyelination. However, the data clearly show that in this model system, the observed effects entirely depend on Fc‐dependent mechanisms, while the variable Fab fragment is not needed to convey protection. These data provide a basis for the continued preclinical evaluation of the variably glycosylated Fc in order to support advancement to clinical studies of neurological disease.

## CONFLICT OF INTEREST

The patent describing the original hexamers is assigned to CSL Behring Lengnau AG. R.J.P. and P.A.B. declare that the molecules discussed within are subject to ongoing patent applications. The other authors have no financial conflicts of interest.

## AUTHOR CONTRIBUTIONS

R.J.P. and N.G. conceived and designed the overall study. C.B., J.S., R.J.P. and P.A.B. designed and performed experiments. V.S. provided binding data shown in Figure S7. J.W. and D.M.C. contributed to the AFM analysis (Figure 5). D.L., A.D. and S.H. contributed to the glycan analysis (Figure S2). A.K., C.L. and V.S. contributed to the raw transcriptomic data (submitted to the European nucleotide archive under accession number PRJEB41654), which was analysed and interpreted by R.J.P. All authors commented on drafts (written by R.J.P.) and reviewed the final manuscript.

## Supporting information

Supplementary Material
