# Supplementary material for: Modified recombinant human IgG1‐Fc is superior to natural intravenous immunoglobulin at inhibiting immune‐mediated demyelination
Source: Immunology. 2021 May 9;164(1):90–105. doi: 10.1111/imm.13341 (PMC8358725; doi:10.1111/imm.13341)
Supplement: Supplementary file 1 — Supplementary Material [file IMM-164-90-s001.pdf]

Fig. S1

A)

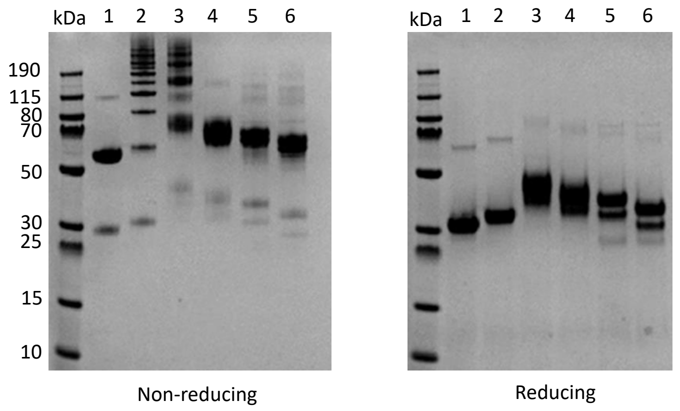

B)

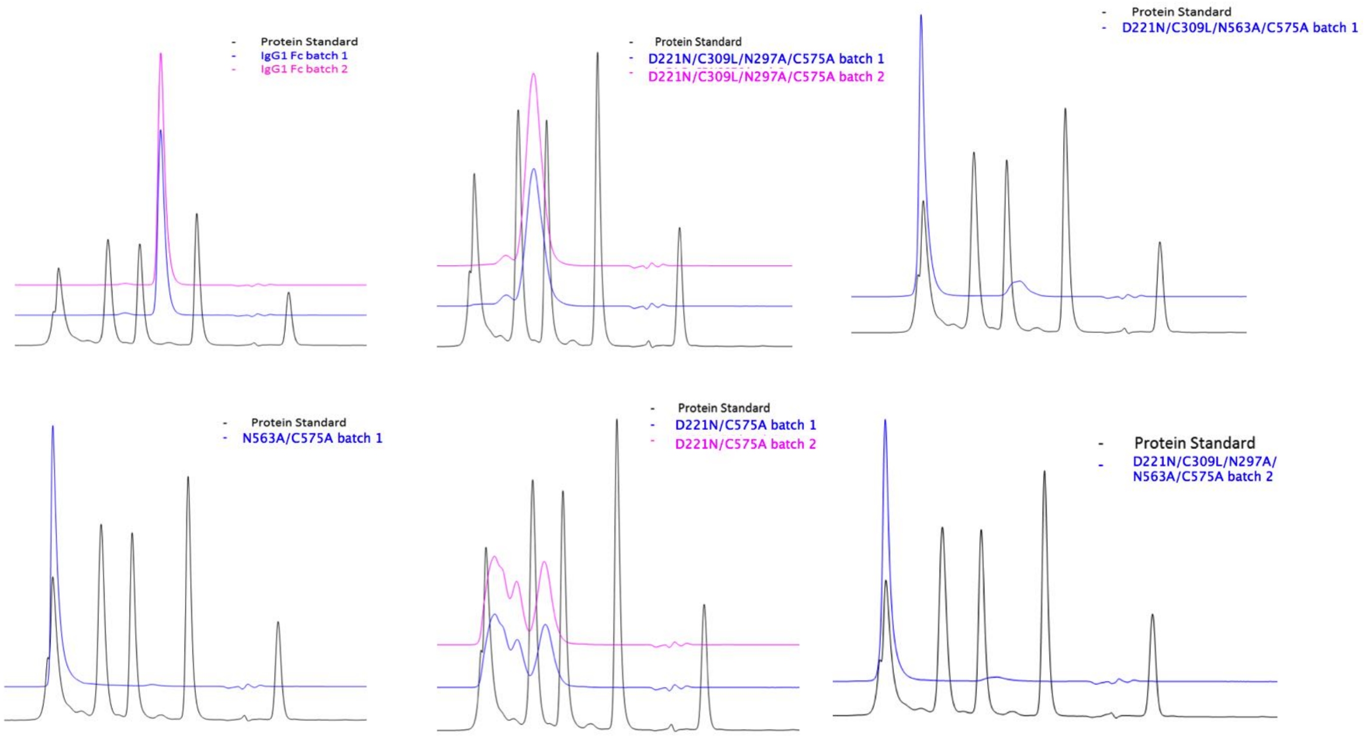

Fig. S1. Characterization of recombinant variably glycosylated Fc proteins. (A) Individual Fc proteins at 2μg was loaded on a 4-12% NuPAGE SDS gel and stained with Coomassie Blue. Pre-stained molecular weight markers (PageRuler Plus (ThermoFisher, Loughborough, UK) are also loaded to allow sizing of the Fc proteins run under non-reducing or reducing conditions. Lane 1, IgG1-Fc wildtype; lane 2, N563A/C575A; lane 3, D221N/C575A; lane 4, D221N/C309L/N297A/C575A; lane 5, D221N/C309L/N563A/C575A; lane 6, D221N/C309L/N297A/N563A/C575A. Wildtype IgG1-Fc runs at the expected molecular weight of ~55 kDa while the variably glycosylated Fc leads are larger as a consequence of the additional C-terminal eighteen amino acid tailpiece and the presence or absence of additional N-linked glycosylation at Asn-221, Asn-297, or Asn-563 (accompanying explanation of structures and location of N-linked glycans are shown in Table 1). **(B) Size analysis of variably glycosylated Fcs using SE-HPLC.** An Acquity UPLC protein BEH SEC 200 Å analytical column (Waters, Hertfordshire, UK) and pre-equilibrated with 0.2 μm filtered PBS. The Fc samples (blue or pink traces dependent on batch) at 10 μg was placed in a pre-cooled auto-sampler at 4°C and injected onto the column using 0.2M Potassium Phosphate buffer, pH 6.8, 0.2M KCl as the mobile phase. Each sample was run for 1.5 column volumes at a flow rate of 0.5 mL/min. Elution was monitored at 280 and 214 nm. The column was calibrated by running standard proteins (BioRad: thyroglobulin, bovine IgG, albumin, myoglobin and uracil) under the same conditions (black traces). Pink and blue traces represent different commercial batches of the same Fc. Summarised in Table 1.

Fig. S2

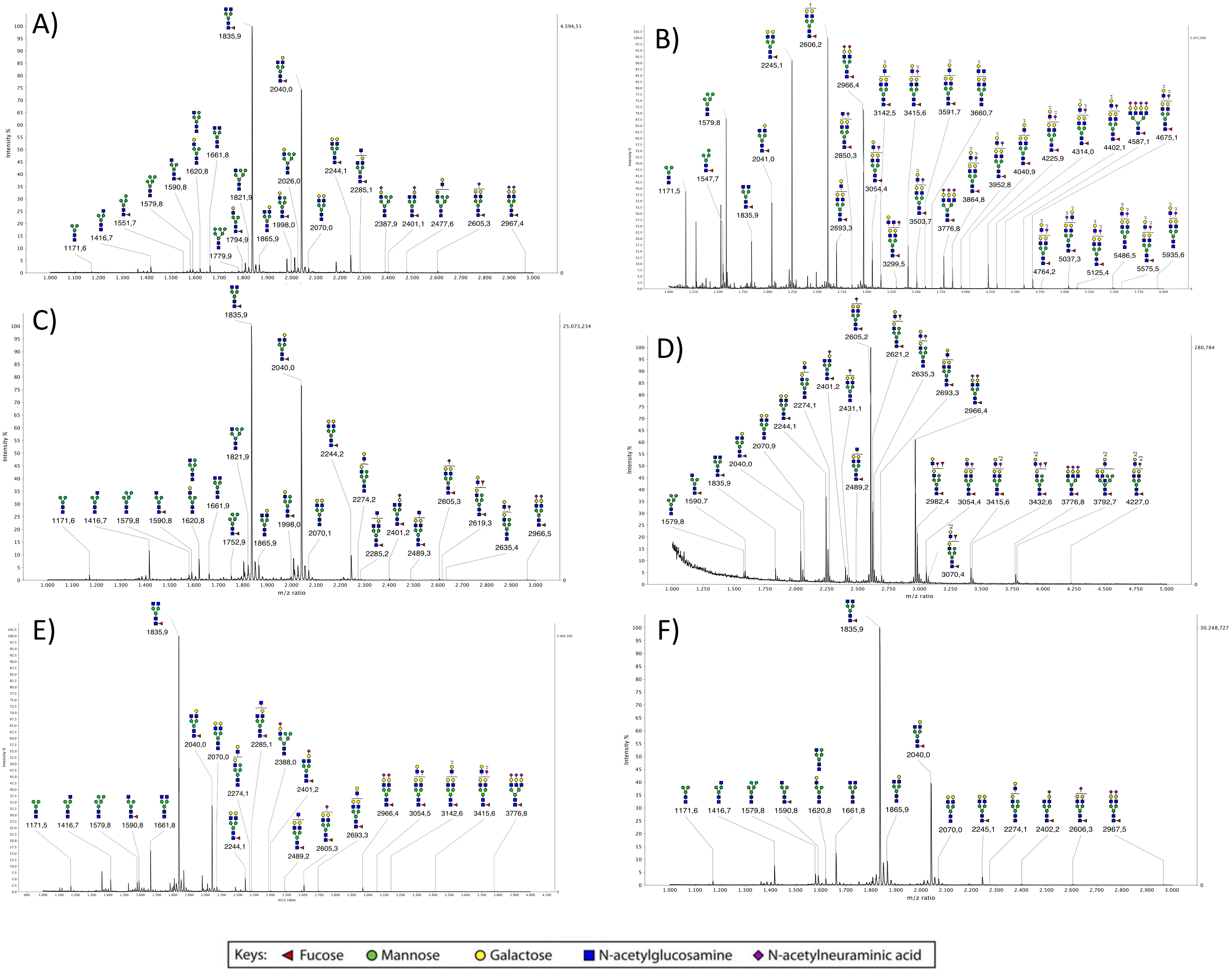

Fig. S2. N-glycomic analysis of Fc mutants. (A) IgG1-Fc, (B), D221N/C309L/N297A/C575A, (C) D221N/C575A, (D) D221N/C309L/N297A/N575A/C575A, (E) D221N/C309L/N563A/C575A, and (F) N563A/C575A. Briefly, the N glycans from 50 µg of each sample was released by incubation with NEB Rapid™ PNGase F and isolated from peptides using Sep-Pak C18 cartridges (Waters) as described previously[1]. The released N-glycans were permethylated, prior to Matrixassisted laser desorption ionization (MALDI) MS analysis. Data were acquired using a 4800 MALDI-TOF/TOF mass spectrometer (Applied Biosystems) in the positive ion mode. The data were analyzed using Data Explorer (Applied Biosystems) and Glycoworkbench[2]. The proposed assignments for the selected peaks were based on composition together with knowledge of biosynthetic pathways.

[1]North, S. J., J. Jang-Lee, R. Harrison, K. Canis, M. N. Ismail, A. Trollope, A. Antonopoulos, P. C. Pang, P. Grassi, S. Al-Chalabi, et al. 2010. Mass spectrometric analysis of mutant mice. Methods Enzymol. 478: 27–77.
[2]Ceroni, A., K. Maass, H. Geyer, R. Geyer, A. Dell, and S. M. Haslam. 2008. GlycoWorkbench: a tool for the computer-assisted annotation of mass spectra of glycans. J. Proteome Res. 7: 1650–1659.

Fig. S3

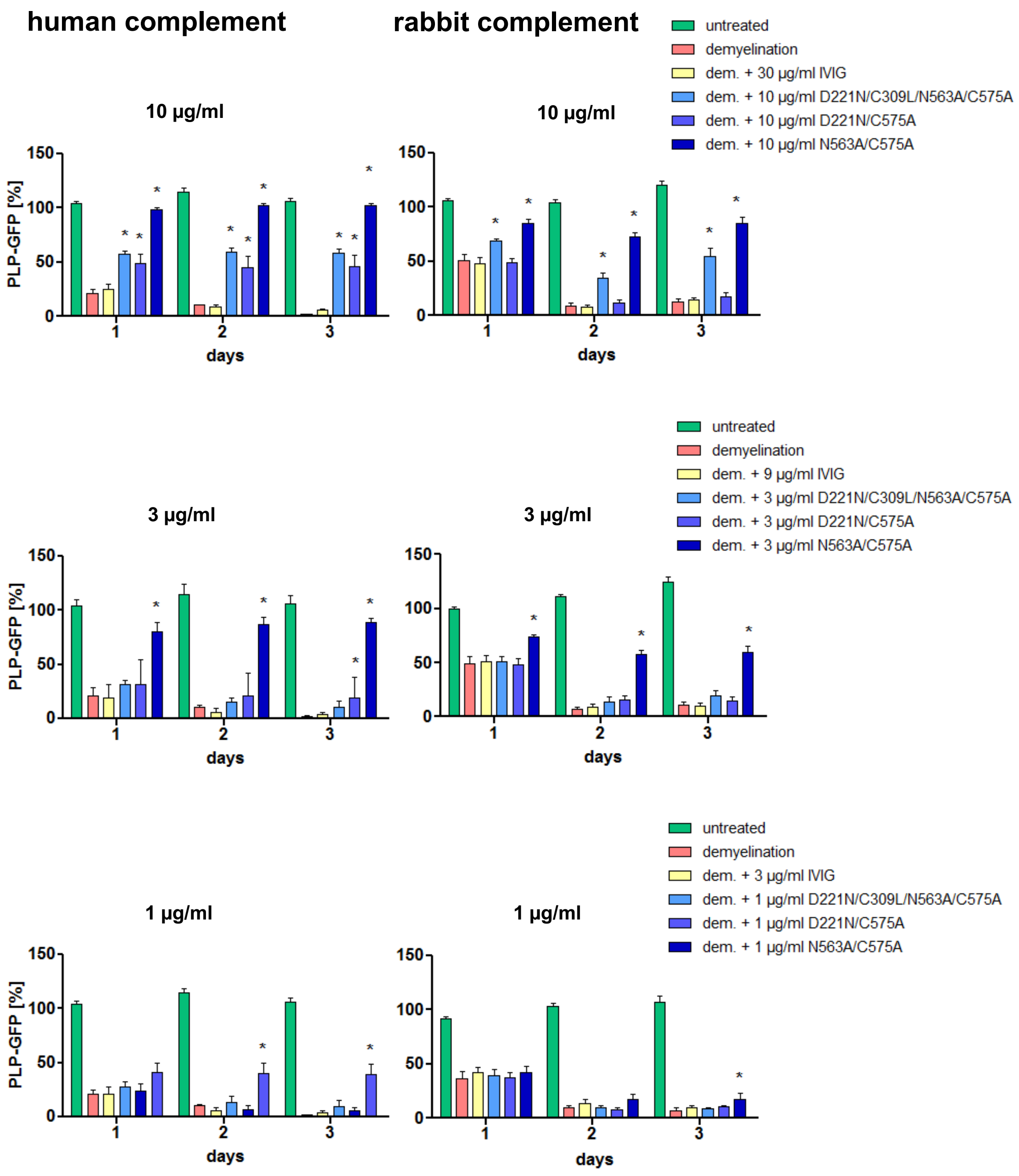

Fig. S3. Glycosylated Fc leads protect from immune-mediated demyelination induced with human complement. OSCs were demyelinated with 5 µg/mL anti-MOG antibody and either 6% rabbit (n = 8-17 OSC per group) or 11% human complement (n = 3-5 OSC per group) for three days in the absence or presence of 1, 3 or 10 µg/mL of three different glycosylated Fc leads (D221N/C309L/N563A/C575A; N563A/C575A and D221N/C57A) or the equimolar amount of WT-IVIG. D221N/C309L/N563A/C575A and N563A/C575A inhibit demyelination at 10 µg/ml independent of the use of human or rabbit complement. D221N/C57A only protected significantly when demyelination was induced with human complement. Demyelination was quantified by GFP fluorescence emitted from myelin of OSCs prepared from transgenic mice expressing GFP under regulatory elements of the myelin protein PLP. Significances were calculated in respect to the demyelinated control using one-way analysis of variance and Dunnett's post hoc test. *P ≤ 0.05. Values are depicted as mean ± SEM.

Figure S4

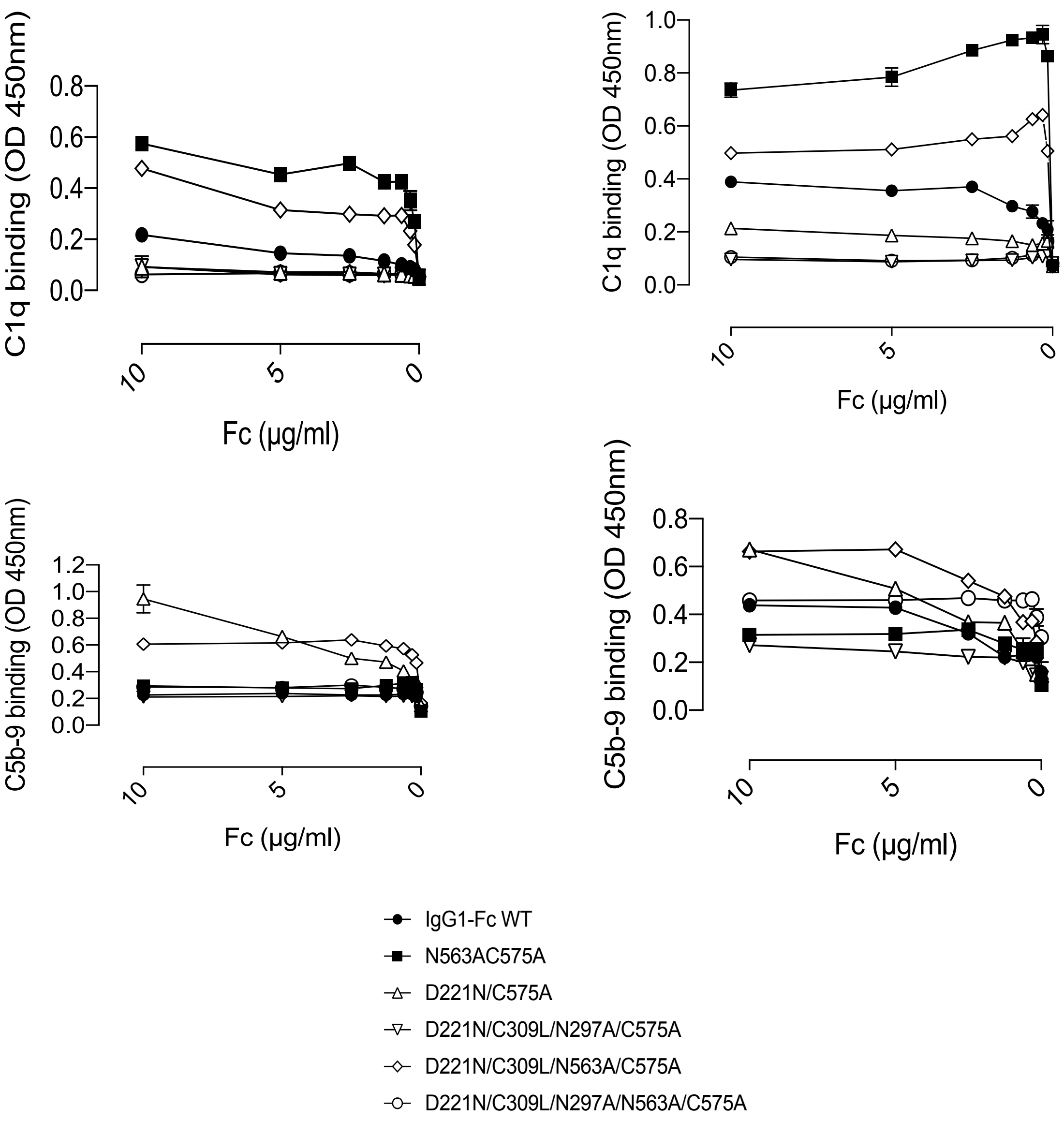

Fig. S4. **Complement binding by Fc mutants.** Complement binding and activation was assessed by ELISA on Fc coated wells as described previously (23-25). Serial dilutions of Fc from 10 µg/ml were coated onto maxi-sorb 96 ELISA plates at 100 µl/well (Fisher Scientific, NC9229197) in 0.05M carbonate buffer (Sigma C3041) overnight at 4°C. Following washing with PBST (PBS-Tween-20 0.05%), plates were incubated with human serum diluted 1/100 in gelatin veronal buffer (Sigma G6514) for 1h at room temperature. After washing in PBST, plates were incubated with either a 1/250 dilution of sheep anti-human C1q-HRP (Serotec: 2221-5004P) or a 1/500 dilution of mouse anti-human C5b-9 mAb (Serotec: MCA2611) in PBST supplemented with 0.5% w/v BSA (Sigma) for 2h. For detection of the C5b-9 binding mAb, plates were washed as before prior to incubation for 2h with a 1:500 dilution of anti-mouse IgG2b-HRP (Southern Biotech: 1090-05). Plates were then washed ten times in PBST before developing for HRP substrate 3,3',5,5'-tetramethyl-benzidine dihydrochloride (Sigma T3405) in phosphate-citrate buffer (Sigma P4922). Reactions were stopped with 25µl 2M H_2SO_4 and plates read on a plate reader at OD450nm. Duplicate experiments are shown.

Figure S5

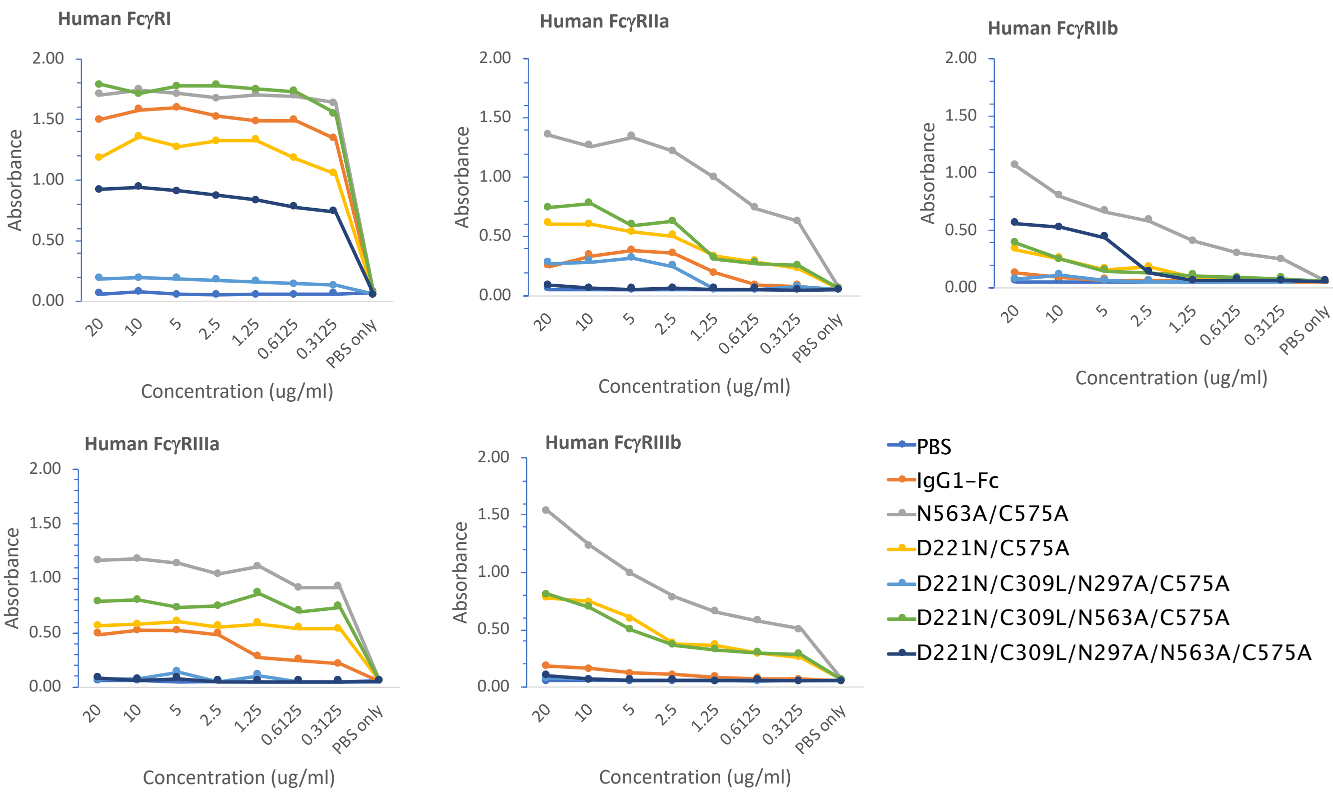

Fig. S5. Binding of variably glycosylated Fc fragments to classical human FcγRs. ELISAs were used to investigate binding of Fc glycan mutants to human FcγRI, FcγRIIA, FcγRIIB, FcγRIIIA, and FcγRIIIB (Bio-Techne). Receptors were coated down on ELISA plates (Nunc) in carbonate buffer pH 9 (Sigma-Aldrich) at 2 μg/ml overnight at 4°C, unless otherwise specified. The plates were blocked in PBS/0.1% Tween-20 (PBST) containing 5% dried skimmed milk. Plates were washed three times in PBST before adding Fc mutant proteins at the indicated concentrations and left at 4°C overnight. Plates were washed as above and incubated for 2h with 1:500 dilution of an alkaline phosphatase-conjugated goat F(ab')$_2$ anti-human IgG (Jackson Laboratories). Binding of the secondary detecting Fab'$_2$ anti-human Fc was checked by direct ELISA to every mutant to ensure there were no potential biases in the detection of binding of different mutants to different receptors. Plates were washed and developed with 100 μl/well of a Sigmafast *p*-nitrophenyl phosphate solution (Sigma-Aldrich). Plates were read at 405nm, and data plotted with GraphPad Prism. n=2 independent experiments.

Figure S6

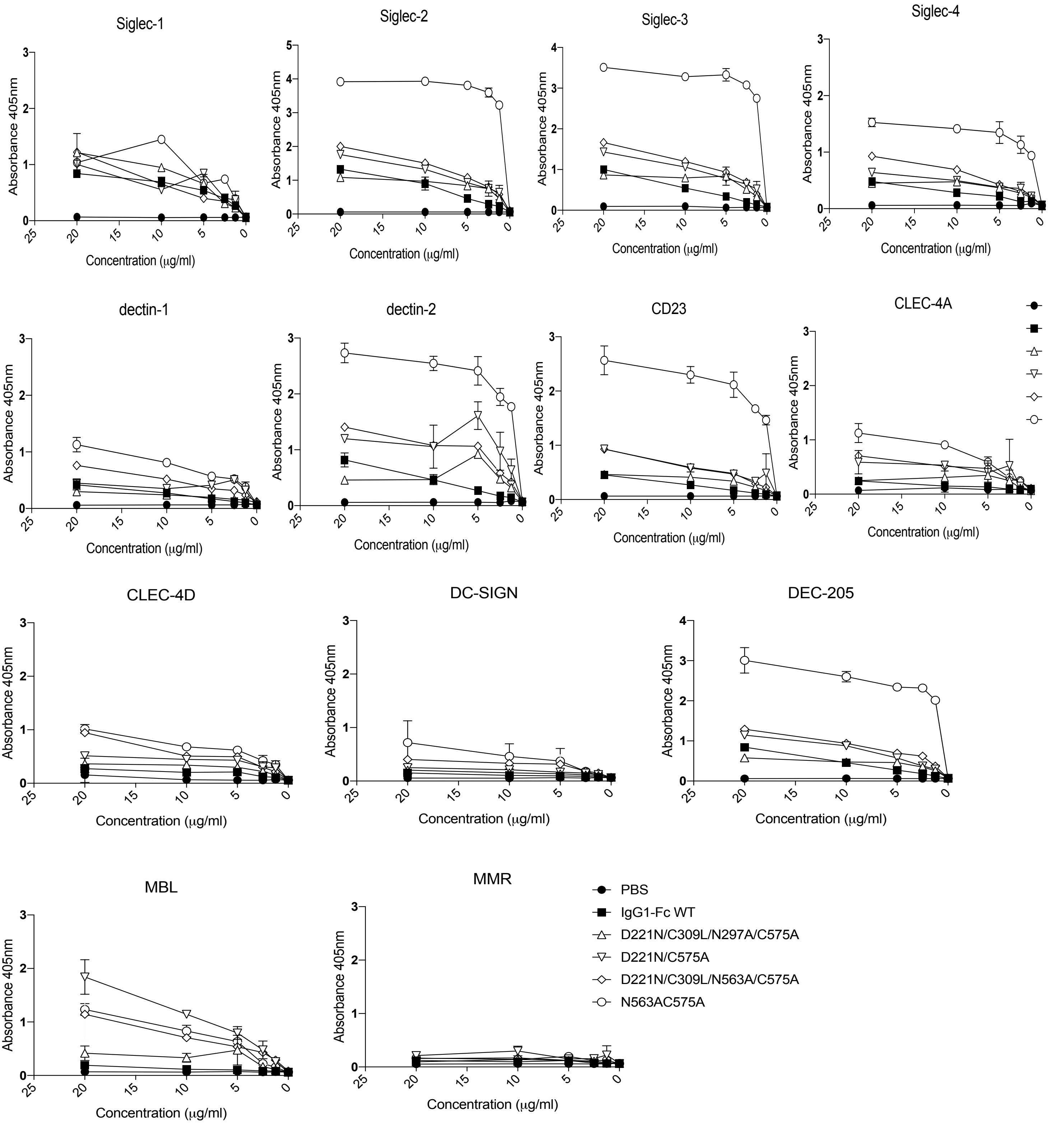

Fig. S6. Binding of variably glycosylated Fc fragments to glycan receptors by ELISA. Methods describing the binding of mutants to tetrameric human DC-SIGN (Elicityl), Siglec-1, Siglec-4, and Siglec-3 (Stratech Scientific), Siglec-2, CD23, dec-1, dec-2, clec-4a, clec-4d, MBL and MMR (Stratech Scientific or Bio-Techne) have been described previously (23-25). Error bars represent SD around the mean; n=2 independent experiments.

Fig. S7

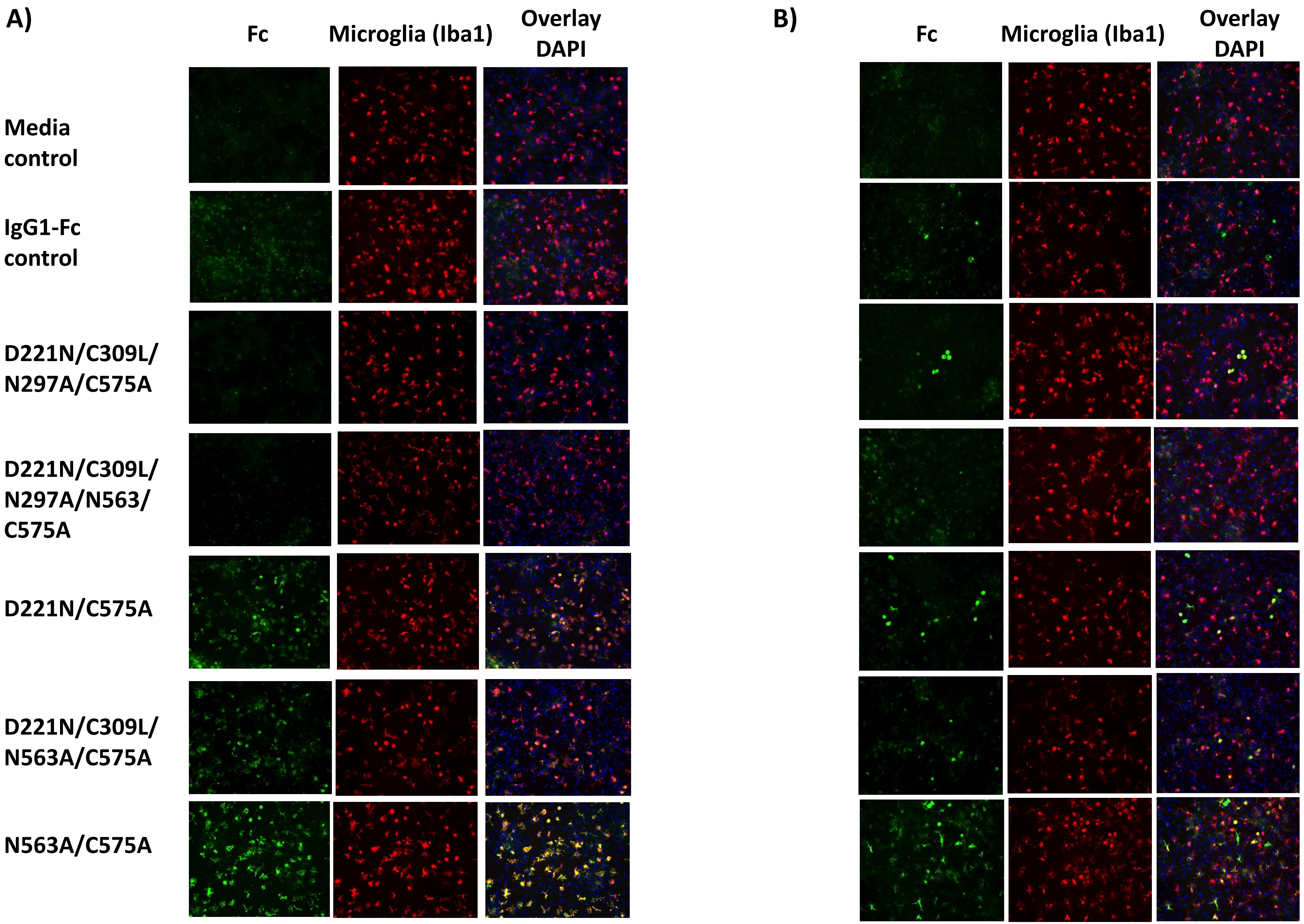
A)
Fc
Microglia (Iba1)
Overlay DAPI
Media control
IgG1-Fc control
D221N/C309L/ N297A/C575A
D221N/C309L/ N297A/N563/ C575A
D221N/C575A
D221N/C309L/ N563A/C575A
N563A/C575A
B)
Fc
Microglia (Iba1)
Overlay DAPI

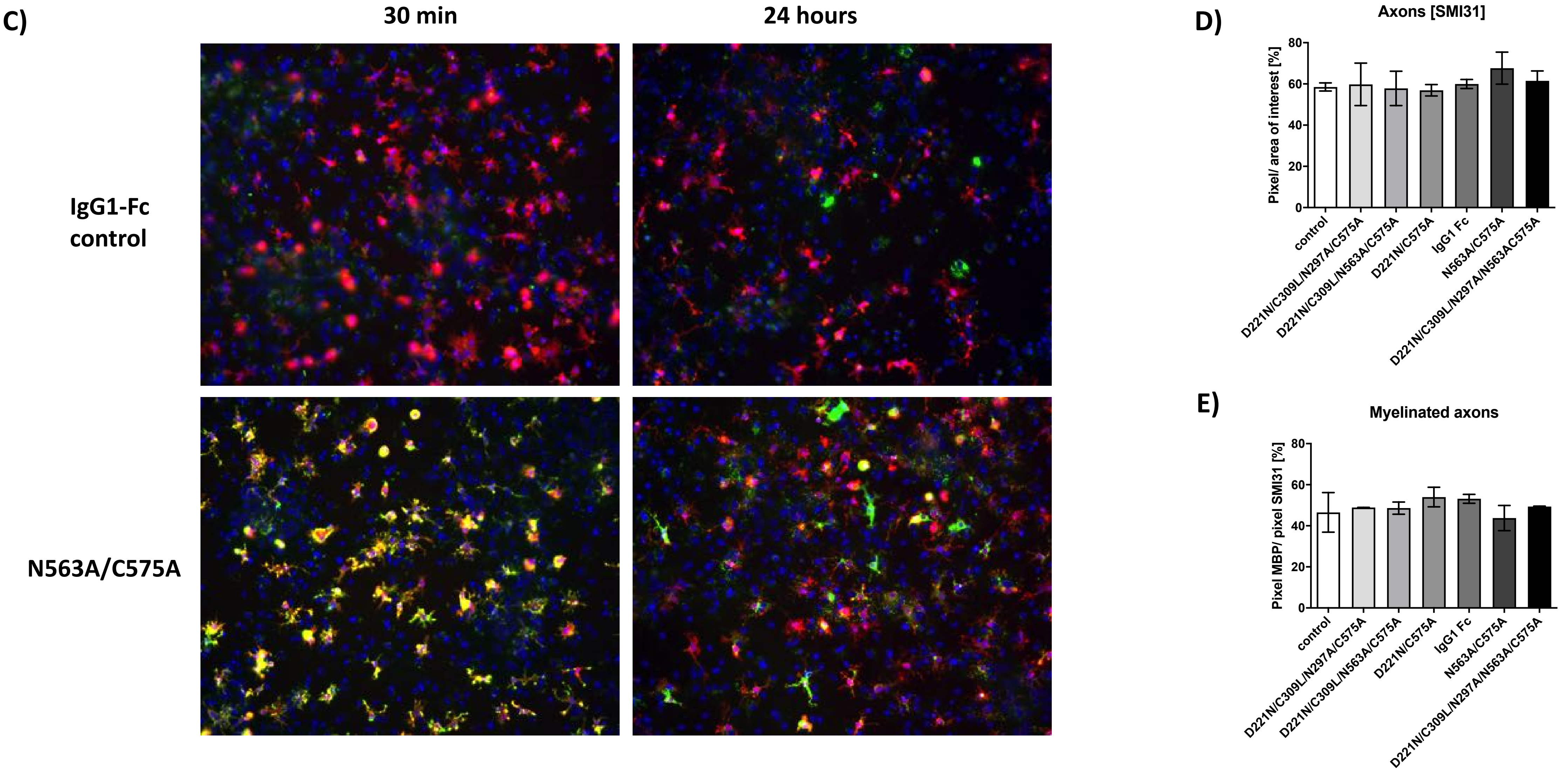

Fig. S7 The antibody fragments bind to microglia in CNS myelinating cultures. Representative images of CNS myelinating cultures (C57Bl6, DIV28) stained for human IgG1 Fcy (green signal), microglia (Iba1, red signal), DAPI (blue signal). The cultures were incubated with the fragments at 4°C for 30 min (panel A) or 24 hours (panel B) and fixed thereafter. (C) High resolution images of overlays from panels A and B above for IgG1-Fc and N563A/C575A. Although not as stark similar findings were observed for D221N/C575A and D221N/C309L/N563A/C575A. The binding to microglia is summarised in Table 1. Quantification of myelin (D) and axonal (E) density after 24 hour incubation using cell profiler software. Treatment of the cultures for 24 hours does not alter myelin and axonal density (N = 2; Mean ± SEM).

Fig. S8.

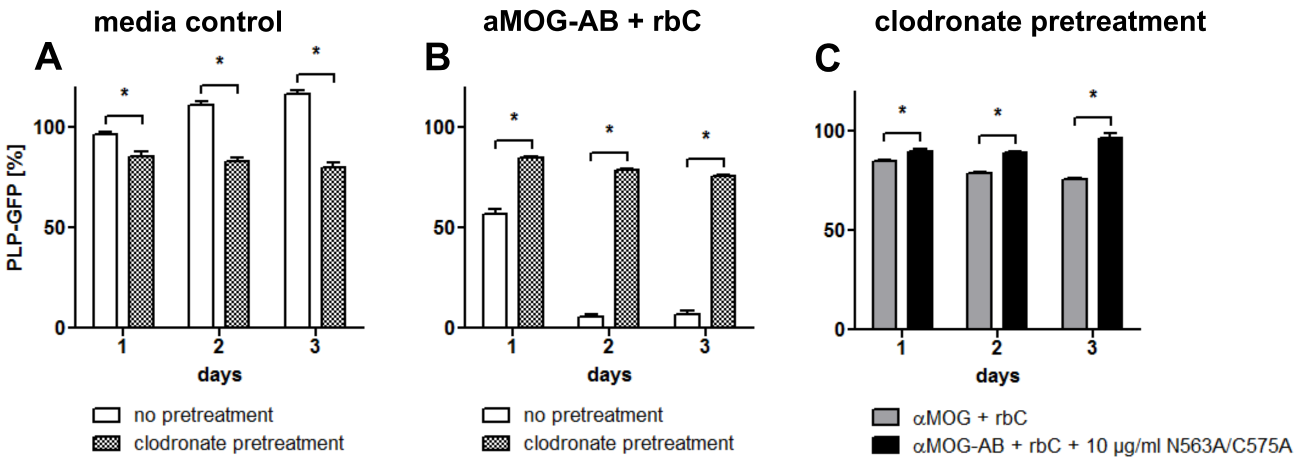

Fig. S8. Microglia depletion via clodronate strongly reduces immune-mediated demyelination. Six days prior to demyelination, OSC was either pre-treated with 300 µg/ml of clodronate (dissolved in H_2O; Sigma-Aldrich, Hamburg, Germany) or left untreated. In both groups, OSC were demyelinated with 5 µg/mL anti-MOG antibody and 8% rabbit complement (rbC) in the presence or absence of 10 µg/mL of the N563A/C575A mutant or were left untreated. Quantification of the GFP+ area relative to day 0 (n = 4-12 OSC per group). Significant differences between treatment groups were calculated with the student's t-test. *P ≤ 0.05. Values are depicted as mean ± SEM. **(A)** While myelination goes on in untreated OSC, this process is reduced by clodronate pre-treatment. **(B)** Clodronate pre-treatment of OSC strongly reduces immune-mediated demyelination. **(C)** Immune-mediated demyelination is further reduced in OSC pre-treated with clodronate and incubation with the N563A/C575A mutant during demyelination.

Fig. S9

A)

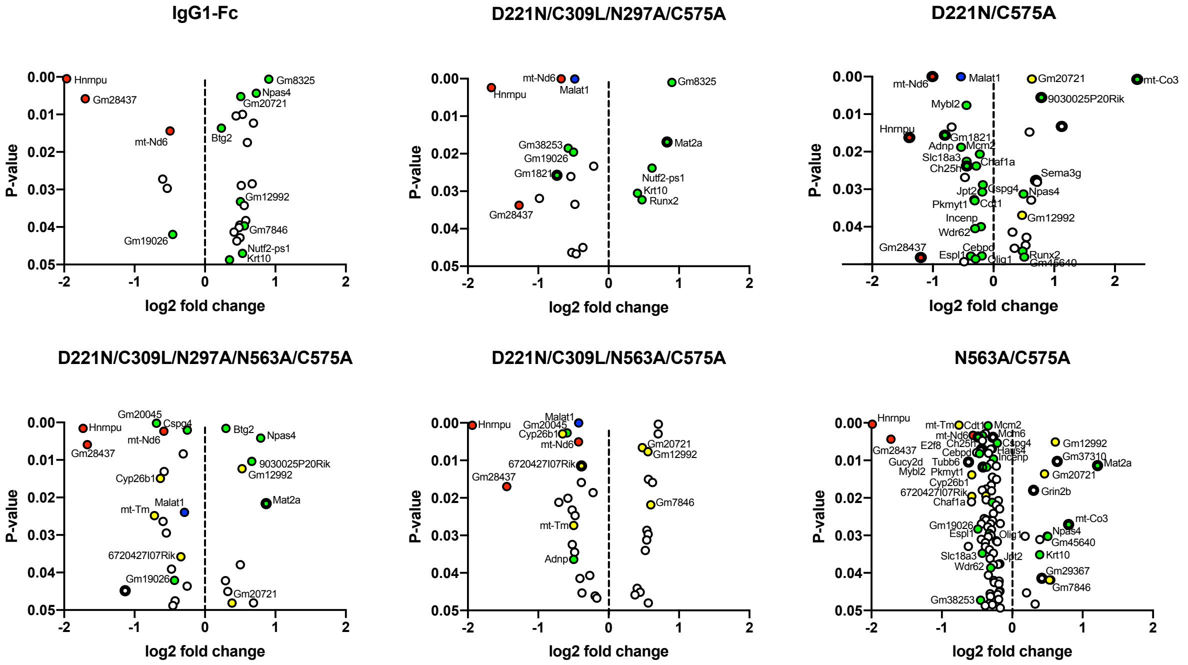

B)

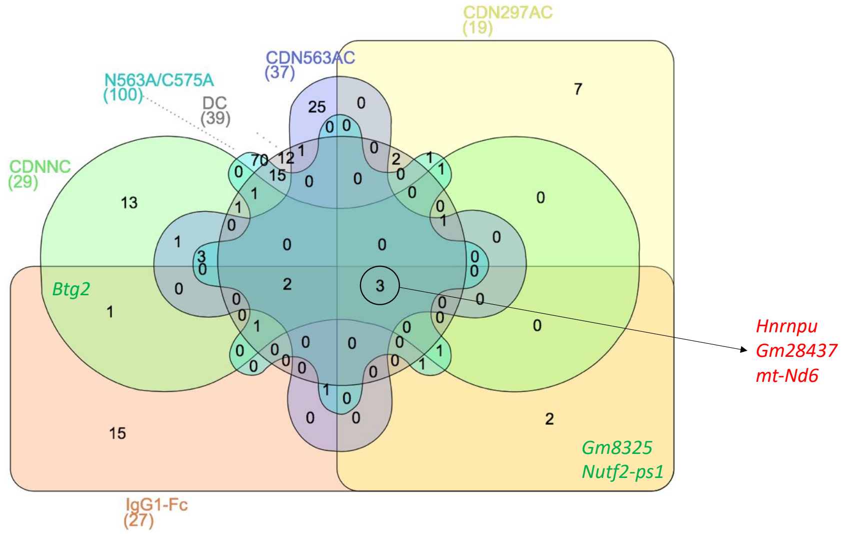

Fig. S9. Transcriptome analysis of leads against media. (A) Individual volcano plots showing differentially expressed genes derived from mouse myelinating CNS after 24h incubation with different indicated Fc mutants. White circles represent unique genes, red circles show genes shared by all the constructs, blue circles show genes shared by 5 constructs, yellow circles show genes shared by 4 constructs, green circles show genes shared with 3 constructs. Thick circles represent genes shared in Fig. S10. All raw data submitted to the European nucleotide archive under accession number PRJEB41654. **(B)** Venn diagram illustrating distribution of high score matches among the six tested Fc compounds. DC = *D221N/C575A*, CDN563AC = *D221N/C309L/N563A/C575A*, CDN297AC = *D221N/C309L/N297A/C575A*, CDNNC = *D221N/C309L/N297A/N563A/C575A.* Methods for transcriptome analysis are provided in the main manuscript.

Fig. S10

A)

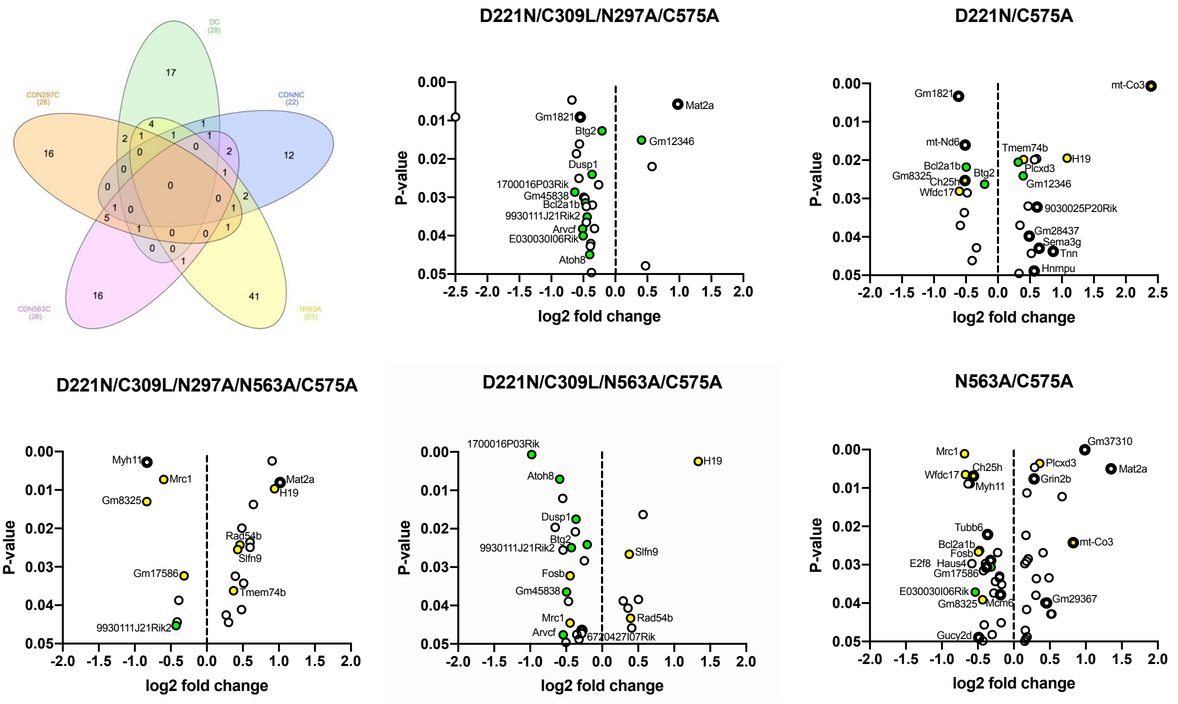

B)

Cellular distribution of differentially expressed genes shared between constructs: https://web.stanford.edu/group/barres_lab/brain_rnaseq.html

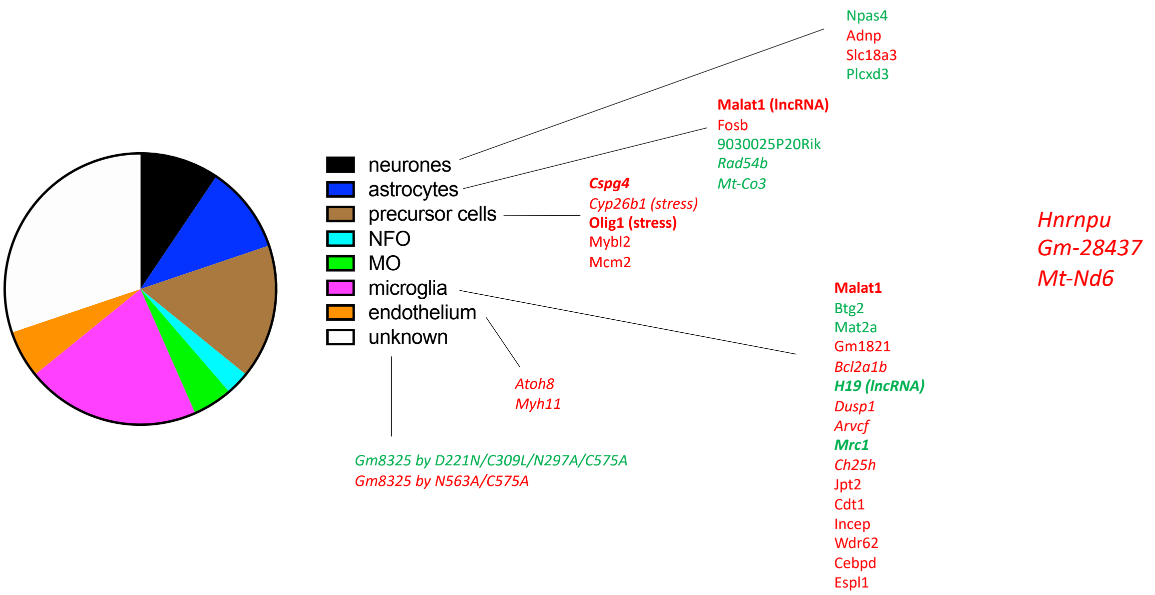

Fig. S10. Transcriptome analysis of leads against IgG1-Fc. (A) Individual volcano plots showing differentially expressed genes derived from mouse myelinating CNS after 24h incubation with different indicated Fc mutants. White circles represent unique genes, yellow circles show genes shared by 3 constructs, green circles show genes shared with 2 constructs. Thick circles represent genes shared in Fig. S9. All raw data the European nucleotide archive under accession number PRJEB41654. Inset a Venn diagram illustrating distribution of high score matches among the five comparator Fc mutants. DC = *D221N/C575A*, CDN563AC = *D221N/C309L/N563A/C575A*, CDN297AC = *D221N/C309L/N297A/C575A*, CDNNC = *D221N/C309L/N297A/N563A/C575A*. **(B)** Cellular distribution of differentially expressed genes shared between constructs. Methods provided in the main text to the manuscript.

Table S1. Summary of Iba1 and CD68 staining experiments described in Fig. 3.

treatment:	Fc only	Fc + complement	Fc + complement + αMOG-Ab
N563A/C575A	No change	Reduction	Reduction
D221N/C309L/N297A/C575A	No change	No change	No change
D221N/C575A	No change	No change	No change
IgG1-Fc	No change	No change	No change
D221N/C309L/N563A/C575A	No change	No change	No change
effect on:	CD68 and Iba1 expression		
